# Modeling the Oxygen Isotope Anomaly (Δ^17^O) of Reactive Nitrogen in the Community Multiscale Air Quality Model: Insights into Nitrogen Oxide Chemistry in the Northeastern United States

**DOI:** 10.1021/acsestair.3c00056

**Published:** 2024-04-22

**Authors:** Wendell W. Walters, Havala O. T. Pye, Heejeong Kim, Meredith G. Hastings

**Affiliations:** † Department of Chemistry and Biochemistry, University of South Carolina, Columbia, South Carolina 29208, United States; ‡ Office of Research and Development, U.S. Environmental Protection Agency, Durham, North Carolina 27703, United States; § Department of Earth, Environment, and Planetary Sciences, Brown University, Providence, Rhode Island 02912, United States; ∥ Institute at Brown for Environment and Society, Brown University, Providence, Rhode Island 02912, United States

**Keywords:** nitrogen oxides, nitrate, air quality, deposition, reactive nitrogen, modeling

## Abstract

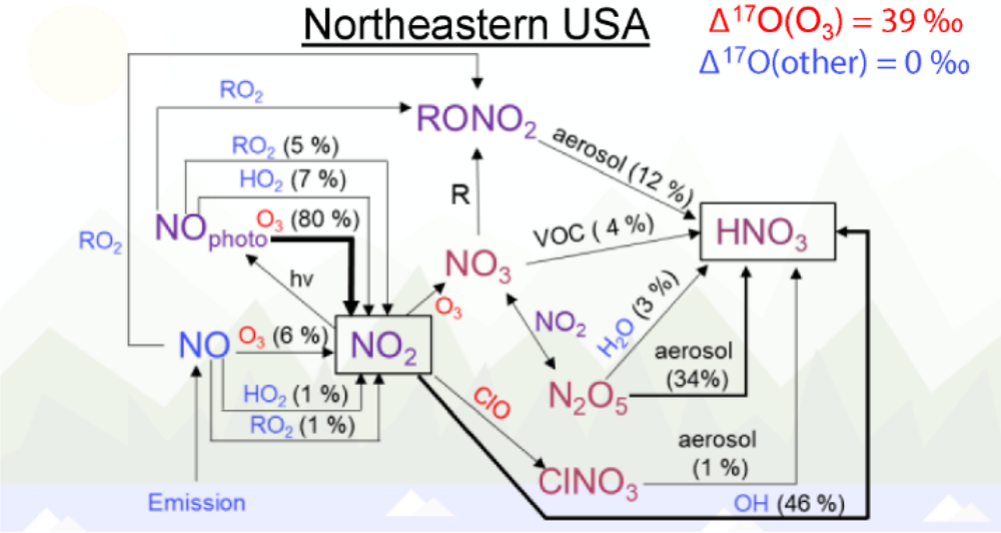

Atmospheric nitrate, including nitric acid (HNO_3_), particulate nitrate (pNO_3_), and organic nitrate (RONO_2_), is a key atmosphere component with implications for air quality, nutrient deposition, and climate. However, accurately representing atmospheric nitrate concentrations within atmospheric chemistry models is a persistent challenge. A contributing factor to this challenge is the intricate chemical transformations involving HNO_3_ formation, which can be difficult for models to replicate. Here, we present a novel model framework that utilizes the oxygen stable isotope anomaly (Δ^17^O) to quantitatively depict ozone (O_3_) involvement in precursor nitrogen oxide (NO_*x*_ = NO + NO_2_) photochemical cycling and HNO_3_ formation. This framework has been integrated into the US EPA Community Multiscale Air Quality (CMAQ) modeling system to facilitate a comprehensive assessment of NO_*x*_ oxidation and HNO_3_ formation. In application across the northeastern US, the model Δ^17^O compares well with recently conducted diurnal Δ^17^O(NO_2_) and spatiotemporal Δ^17^O(HNO_3_) observations, with a root mean square error between model and observations of 2.6‰ for Δ^17^O(HNO_3_). The model indicates the major formation pathways of annual HNO_3_ production within the northeastern US are NO + OH (46%), N_2_O_5_ hydrolysis (34%), and organic nitrate hydrolysis (12%), with significant seasonal variability. This model can evaluate NO_*x*_ chemistry in CMAQ in future air quality and deposition studies involving reactive nitrogen.

## Abstract 2

An innovative model framework integrates the oxygen stable isotope anomaly to assess NO_*x*_ chemistry in Community Multiscale Air Quality (CMAQ), revealing significant spatiotemporal patterns and establishing a new tool for evaluating tropospheric oxidation chemistry.

## Introduction

Nitrogen oxides (NO_*x*_), encompassing nitrogen oxide (NO) and nitrogen dioxide (NO_2_), are important trace gases from human and biogenic activities that play a significant role in air quality, nutrient deposition, and climate change.^1−3^ NO_*x*_ affects the atmosphere’s oxidation capacity, influencing various trace gas concentrations.^4^ Ultimately, NO_*x*_ oxidizes into atmospheric nitrate, with inorganic nitrate (nitric acid (HNO_3_) and particulate nitrate (pNO_3_)) dominating globally and organic nitrates (RONO_2_) being relevant in rural continental areas.^5^ In the US, regulations have reduced NO_*x*_ emissions from vehicles and power plants,^6,7^ yet nitrogen deposition from the atmosphere remains a key environmental stressor, impacting land, water quality, and climate interactions.^8^

Atmospheric chemistry and transport models are commonly used to predict air quality and the implications of environmental policies.^9,10^ However, these models struggle to accurately replicate observed HNO_3_ and inorganic pNO_3_ concentrations, including magnitude, seasonality, and urban–rural gradients, posing challenges for predicting air quality under policy change (e.g., the Clean Air Act Secondary Standards^11,12^). The disagreement between model and observations may be related to complicated production mechanisms of HNO_3_ driven by nonlinear chemical feedbacks, heterogeneous chemistry, competing inorganic and organic pathways, and gas/particle partitioning.^13,14^ Indeed, uncertainties in the rate of NO_*x*_ oxidation to HNO_3_ also contribute to uncertainties in the formation of significant atmospheric oxidants like ozone (O_3_) and hydroxyl radicals (OH).^15^ As such, enhancing our understanding of NO_*x*_ chemistry representation in atmospheric models is crucial for estimating nitrogen deposition and comprehending greenhouse gas lifetimes, in addition to predicting air quality.

The stable oxygen isotope anomaly (Δ^17^O = δ^17^O – 0.52 × δ^18^O) has emerged as a promising tool to study NO_*x*_ photochemical cycling and atmospheric nitrate formation.^16−21^ This is because the transferable O atoms of O_3_ at its terminal ends contain an elevated Δ^17^O(O_3_^term^) of (39 ± 2‰),^22^ while all other major atmospheric oxidants including OH, water vapor, and peroxy radicals (HO_2_ and RO_2_) have a Δ^17^O near 0‰.^23,24^ This enables quantitative insights into the role of O_3_ in NO_*x*_ photochemistry and nitrate formation pathways. For example, numerous studies have documented a large seasonal change in Δ^17^O from HNO_3_ and pNO_3_ collected in rainwater and aerosol studies, reflecting a seasonal chemical shift from O_3_ in winter to HO_*x*_/RO_2_ during summer.^12,16,24−26^ Additionally, the elevated Δ^17^O values of atmospheric nitrate serve as a useful tracer for evaluating nitrate source contributions in ecosystem studies.^27−29^

However, to take full advantage of the diagnostic value of Δ^17^O observations, a model framework that can be used to interpret NO_*x*_ oxidation chemistry and potentially provide insights into its connection to nitrogen deposition is required. Several 0-D box models have been developed to incorporate Δ^17^O tracers to assess oxidation chemistry.^16,30^ While these relatively simplistic models have reasonably matched Δ^17^O observations, they require several assumptions regarding emissions/background concentrations and aerosol chemistry. These models are unsuitable for evaluating model representation of NO_*x*_ chemistry and deposition of its secondary products that require a more sophisticated treatment of emissions, chemistry, transport, and meteorology. Currently, only one global atmospheric chemistry transport model is developed to incorporate Δ^17^O as a tracer of nitrate chemistry (GEOS-Chem).^31,32^ This model does a reasonable job simulating coarse Δ^17^O features of global HNO_3_ production. For example, the global Δ^17^O model (resolution = 4° × 5°) of HNO_3_ was reported to have an *R*^2^ of 0.51 relative to a compilation of global observations.^32^ However, large uncertainties remain in how well atmospheric models simulate regional HNO_3_ production at a higher-resolution spatial scale,^12^ which are particularly relevant for air quality and deposition-related studies. Further, the current model framework may suffer from oversimplifications in its treatment of NO_*x*_ photochemical equilibrium assumptions, which has been pointed out as a potentially large source of uncertainty.^32^ Recent oxygen isotope observations of NO_2_ have indicated the importance of incomplete NO_*x*_ photochemical cycling inducing diurnal Δ^17^O (and δ^18^O) variabilities;^20,33^ however, this is not considered in current 3D model frameworks. Therefore, establishing another model, particularly one with high spatiotemporal resolution routinely used for atmospheric deposition and air quality-related analyses, with this Δ^17^O tracer vetted against detailed observations would provide an advanced new tool for the atmospheric chemistry community.

In this study, a novel model framework was developed to simulate Δ^17^O of NO_2_ and HNO_3_ in the Northeastern US using the Community Multiscale Air Quality (CMAQ) Model. The northeastern US is a crucial region to study due to its dense population, historical air quality issues, high nitrate deposition, and coexistence of urban and sensitive ecosystems.^34^ Further, this has been a region in which there are large model biases in atmospheric nitrate concentrations relative to observations.^11^ The study aimed to evaluate CMAQ’s portrayal of NO_*x*_ photochemistry and HNO_3_ formation patterns in this region.

## Materials and Methods

### Model Description

The conducted model simulations were based on replicated runs from the US EPA’s Air QUAlity TimE Series Project (EQUATES).^35,36^ Briefly, model simulations were conducted using CMAQv5.4 utilizing Carbon Bond 6, revision 3 with aerosol 7 (cb6r3-ae07) chemical mechanism including the addition of chlorine chemistry as implemented in CMAQ.^37−39^ The model simulations included bidirectional ammonia (NH_3_) exchange,^40,41^ the Surface Tiled Aerosol Gaseous Exchange (STAGE) dry deposition,^37,42−45^ Weather Research and Forecasting (WRF) model version 4.1.1 meteorology,^46^ and emission inputs based on the EQUATES methods, which are largely based on the 2017 national emissions inventory (NEI) with updates by sector and appropriately scaled for the simulation year.^35,35,47,48^ Model simulations were conducted for the northeastern US domain with a resolution of 12 × 12 km within the northern hemisphere boundary conditions for 2015. Boundary and initial conditions for the northeast US domain were obtained from CMAQ simulations performed as part of EQUATES, which is a continuous set of CMAQ simulations over both the northern hemisphere (108 km horizontal resolution) and contiguous U.S. (12 km horizontal resolution) starting December 2001 and continuing through 2019. Initial conditions for the northeast domain (12 km horizontal resolution) for the specific year in this study were obtained from the continuous set of contiguous US simulations at 12 km horizontal resolution. To create the boundary conditions for the northeast US domain, 90 species (including ozone, major nitrogen species, certain VOCs, and fine aerosols) available from the 12 km contiguous US 3D concentration fields were used and any species not available from the contiguous US simulations (e.g., coarse aerosols and select VOCs) were obtained from the 108 km hemispheric simulation. This hybrid boundary condition patching was needed because full 3D species output was not saved on the contiguous US domain. Boundary conditions were specified by hourly values. Simulations in this work used CMAQv5.4, which is a later version than CMAQv5.3 used in the original EQUATES time series. However, we used the same gas- and aerosol-phase chemistry (Carbon Bond 6, revision 3 with aero 7, cb6r3-ae07) to match EQUATES. In addition, we selected an aerosol deposition scheme, STAGE, that produces similar results in CMAQv5.3 and v5.4. No meaningful differences in chemistry are expected compared to EQUATES. The model was conducted with a spin-up time of 16 days.

The cb6r3-ae07 mechanism was modified to explicitly track NO_*x*_ photochemical cycling, which has been shown to have an important impact on the oxygen isotope composition of NO_2_.^20,33^ The modified mechanism was termed cb6r3-ae07-NPC (NO_*x*_ Photochemical Cycle). This modified mechanism tracks whether NO derives from primary emissions (referred to as NO_source_ with respect to its oxygen isotope composition) or from NO_2_ photolysis (NO_photo_). Tracking the type of NO is important for modeling Δ^17^O as the NO_source_ should have a Δ^17^O near 0‰, while NO_photo_ derived from NO_2_ photolysis will have a Δ^17^O that reflects the oxidants involved in NO_*x*_ photochemical cycling. Briefly, NO produced via NO_2_ photolysis was tagged as NO_photo_, indicating that the oxygen isotopes of NO were photochemically cycled with oxidants.^49^ In the cb6r3-ae07-NPC mechanism, all the base model reactions involving NO as a reactant were replicated to include NO_photo_, and NO was reserved for primary emissions of NO that carry an O atom from source emissions with an assumed Δ^17^O(NO) = 0‰ (see below). In the modified mechanism, both NO and NO_photo_ participate in identical reactions, but the concentrations and formation rates, which are utilized to simulate Δ^17^O, are tracked separately for each. Overall, the modified cb6r3-ae07-NPC mechanism included one reaction with adjusted products (NO_2_ photolysis) and 27 reactions replicated for NO_photo_ relative to the cb6r3-ae07 base mechanism. A summary of the modified reactions in the cb6r3-ae07-NPC mechanism is provided in Table S1.

The production rates of NO_2_ and HNO_3_ formation were quantified using Integrated Reaction Rate (IRR) analysis within the Process Analysis (PA) script in CMAQ.^50^ The IRR analysis enables a computationally efficient way to output the individual chemical reaction rates of chemical reactions and species cycling in a specific grid-cell within the model output. The major NO_2_ and HNO_3_ production pathways in cb6r3-ae07-NPC (Table 1) were tagged using IRR, and output was generated as a function of latitude, longitude, atmospheric layer, and time with a resolution of 1 h in the output files. Based on the IRR output, the fractional production (*f*) of the considered NO_2_ (R1-R8; Table 1) and HNO_3_ (R9–R16; Table 1) formation pathways were calculated as a function of latitude, longitude, height, and time:
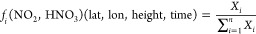
1where *f* represents the fractional production for NO_2_ or HNO_3_, *X* refers to the production amount, and *i* represents the distinct production pathway. The *f*_*i*_ values were calculated based on the production pathways of NO_2_ and HNO_3_ for the lowest 15 atmospheric layers in CMAQ, corresponding to approximately the lowest 1,000 m in the atmosphere, consistent with the expectation that the majority of HNO_3_ is formed in the lower atmosphere.^32^ The *f*_*i*_ values were calculated every hour when simulating diurnal Δ^17^O(NO_2_) variabilities. Additionally, the hourly *f*_*i*_ values were averaged over 24 h to calculate daily Δ^17^O(NO_2_) and Δ^17^O(HNO_3_) values, reflecting the production of NO_2_ and HNO_3_ at a particular grid cell over a 24 h period.

**Table 1:** Summary of the NO_2_ and HNO_3_ Formation Pathways in the cb6r3-ae07-NPC Mechanism, Oxygen Mass Balance, Δ^17^O Transfer Factors for Each Reaction Pathway, and the Modeled Fractional Production for Each Pathway for the Northeastern US Domain for January (Jan), July (Jul), and Annual (Yr) in 2015^a^

rxn	pathway	oxygen mass balance	Δ^17^O transfer factor	*f* (Jan/Jul/yr)
NO_2_				
R1	NO + O_3_	(1/2)(NO_source_) + (1/2)(O_3_)	(1/2)Δ^17^O(O_3_^term^)	0.04/0.10/0.06
R2	NO + HO_2_	(1/2)(NO_source_) + (1/2)(HO_2_)	0	0.00/0.02/0.01
R3	NO + RO_2_	(1/2)(NO_source_) + (1/2)(RO_2_)	0	0.00/0.02/0.01
R4	NO + ClO	(1/2)(NO_source_) + (1/2)(ClO)	(1/2)Δ^17^O(O_3_^term^)	0.00/0.00/0.00
R5	NO_photo_ + O_3_	O_3_	Δ^17^O(O_3_^term^)	0.92/0.61/0.80
R6	NO_photo_ + HO_2_	HO_2_	0	0.03/0.12/0.07
R7	NO_photo_ + RO_2_	RO_2_	0	0.01/0.13/0.05
R8	NO_photo_ + ClO	ClO	Δ^17^O(O_3_^term^)	0.00/0.00/0.00
HNO_3_				
R9	NO_2_ + OH	(2/3)(NO_2_) + (1/3)(OH)	(2/3)Δ^17^O(NO_2_)	0.30/0.58/0.46
R10	NO_3_ + HC	(2/3)(NO_2_) + (1/3)(O_3_)	(2/3)Δ^17^O(NO_2_) + (1/3)Δ^17^O(O_3_^term^)	0.02/0.06/0.04
R11	N_2_O_5_ + H_2_O(g)	(2/3)(NO_2_) + (1/6)(O_3_) + (1/6)H_2_O	(2/3)Δ^17^O(NO_2_) + (1/6)Δ^17^O(O_3_^term^)	0.04/0.02/0.03
R12	N_2_O_5_ + H_2_O(aerosol)	(2/3)(NO_2_) + (1/6)(O_3_) + (1/6)H_2_O	(2/3)Δ^17^O(NO_2_) + (1/6)Δ^17^O(O_3_^term^)	0.59/0.06/0.34
R13	NO_2_ + H_2_O(aerosol)	(2/3)(NO_2_) + (1/3)(H_2_O)	(2/3) Δ^17^O(NO_2_)	0.00/0.00/0.00
R14	ClNO_3_ + H_2_O(aerosol)	(2/3)(NO_2_) + (1/3)(O_3_)	(2/3)Δ^17^O(NO_2_) + (1/3)Δ^17^O(O_3_^term^)	0.02/0.01/0.01
R15	RONO_2_ + H_2_O(aerosol) (from RO_2_ + NO)	(1/3)(NO_2_) + (2/3)(O_2_)	(1/3)Δ^17^O(NO_2_)	0.00/0.08/0.02
R16	RONO_2_ + H_2_O(aerosol) (from R + NO_3_)	(2/3)(NO_2_) + (1/3)(O_3_)	(2/3)Δ^17^O(NO_2_) + (1/3)Δ^17^O(O_3_^term^)	0.03/0.19/0.10

a The Δ^17^O values of NO_source_, HO_2_, RO_2_, OH, and H_2_O are assumed to be 0‰.

Δ^17^O(NO_2_) was calculated based on the NO_2_ relative formation pathways, utilizing oxygen-isotope-mass balance, and assumptions regarding Δ^17^O transfer and values of atmospheric oxidants (eq 2):^24,32^
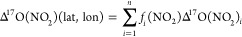
2where Δ^17^O(NO_2_)_*i*_ is the Δ^17^O(NO_2_) value for the particular formation pathway (*i*), calculated based on O-isotope mass balance (Table 1). In a similar manner, the Δ^17^O(HNO_3_) values are then calculated based on the HNO_3_ fractional formation pathways (*f*) and assumed oxygen isotope mass balance (Table 1) (eq 3):
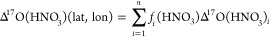
3

In the Δ^17^O(NO_2_) and Δ^17^O(HNO_3_) calculations, Δ^17^O(O_3_^term^) was assumed to be 39‰, which represents the average of near-surface observations derived from O_3_ oxidation of nitrite coated filters (39 ± 2‰)^22^ and is similar to the steady-state value of Δ^17^O(NO_2_) from experimental investigation of NO_*x*_/O_3_ cycling of (39.3 ± 1.9‰).^49^ The Δ^17^O values of RO_2_, HO_2_, OH, and H_2_O were assumed to be 0‰^23,24^ and follow previous Δ^17^O modeling work involving tropospheric chemistry.^31,32^ Additionally, Δ^17^O(NO_source_) was set to 0‰, which should be a reasonable assumption since most NO emissions in the polluted mid-latitudes derive from high-temperature combustion processes involving oxidation from atmospheric O_2_, which has a Δ^17^O value near 0‰.^51^

Overall, the implicit approach of tracking the production rates of NO_2_ and HNO_3_ at a reaction-specific level for offline calculation of Δ^17^O of NO_2_ and HNO_3_ is similar to the approach utilized in GEOS-Chem for global modeling,^31,32^ with the key exception that NO_*x*_ photochemical Δ^17^O equilibrium with oxidants is assessed by directly tracking NO_2_ production from NO that derived from either photolyzed NO_2_ or direct NO emissions in the modified cb6r3-ae07-NPC mechanism. The benefit of tracking production rates rather than concentrations is that it reduces the computational complexity and model run times. For example, developing a chemical mechanism to separately track the ^16^O, ^17^O, and ^18^O isotopes of all compounds would substantially increase the number of species and reactions. Additionally, it reduces the impact of boundary conditions and transport outside the model domain on the simulated isotope values. The main limitation of the rate tracking approach is that it does not account for transport since it only considers the local NO_2_ or HNO_3_ production within each grid cell. However, this limitation should not limit our interpretation of NO_*x*_ oxidation chemistry via comparison to Δ^17^O observations if regional and vertical production trends are similar. Further, tracking the local NO_*x*_ and atmospheric nitrate production has the benefit of providing insight to local production trends versus regional signals when compared to observations. Comparison of observed and modeled Δ^17^O is the focus of our model simulations. The comparison of concentration data of reactive species is beyond the scope of this work; however, we note that the model simulations were based on the US EPA EQUATES simulations, which have shown excellent agreement with reactive nitrogen and sulfur species.^36^

### Δ^17^O Observations in the Northeastern US

**Figure 1:**
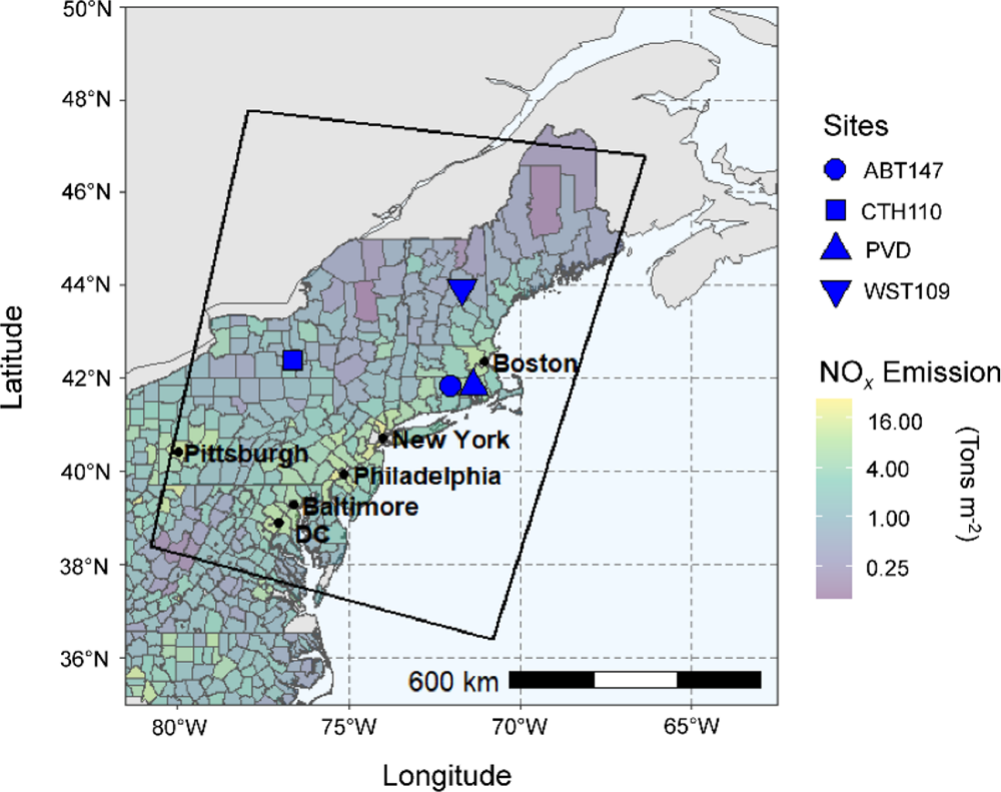
Overview of the northeastern US domain, including the NO_*x*_ emission density (tons m^–2^) for the US counties in the northeastern US based on the US EPA National Emission Inventory 2017. The CMAQ model domain is indicated by the black rectangle. The locations of the various Δ^17^O observations are shape-coded and include Δ^17^O(HNO_3_) observations from CASTNET sites (ABT147, CTH110, WST109), and Δ^17^O(NO_2_) observations from a chemical speciation network site downwind of Providence, RI (PVD).

The model Δ^17^O simulations were compared with new observations of Δ^17^O(NO_2_) and previously reported Δ^17^O(HNO_3_) in the northeastern US.^12^ The location of these monitoring locations and an overview of the northeastern modeling domain, including estimated NO_*x*_ emission density from the 2017 US EPA National Emission Inventory by county, are shown in Figure 1.

### Urban Δ^17^O(NO_2_) Diel Cycle

The model simulations of diel Δ^17^O(NO_2_) values were compared with observations made in Rumford, RI (41.84 °N, 71.36 °W) from March 19 to March 24, 2018. The location of the sampling site was operated by the Rhode Island Department of Health (RI-DOH) and Rhode Island Department of Environmental Management, which is part of the air quality monitoring locations throughout the state. Additionally, this site is co-located with the US EPA Chemical Speciation Network and has been sited to reflect the downwind emissions of the greater metropolitan region of Providence, RI. Ambient NO_2_ was collected using coated honeycomb denuders housed in a ChemComb Speciation Cartridge, as previously described.^52^ Briefly, the honeycomb denuders were coated with 10 mL of 10% NaOH + 25% guaiacol in an 80:20 methanol to water (w/v) solution, which collects NO_2_ as nitrite (NO_2_^–^).^33,52,53^ Air was sampled at a flow rate of 10 standard liters per minute using a mass-flow controlled (Dakota Instruments 6AGC1AL55-10AB) vacuum pump (Welch Vacuum Pump 2522B-01). Samples were collected over 6 h intervals that included 0:00–6:00, 6:00–12:00, 12:00–18:00, and 18:00–0:00 (local time; UTC-4:00) on the rooftop of the air monitoring station. In total, 12 NO_2_ samples were collected for Δ^17^O analysis. Immediately after collection, denuders were separately extracted in 30 mL of ultra-high purity water (18.2 MΩ). The KOH/guaiacol extraction solution was immediately spiked with 1 mL of 10 M NaOH to ensure pH > 13 to prevent O isotopic exchange between NO_2_^–^ and H_2_O_(l)_.^54^ Samples were then frozen until subsequent analysis.

The denuder extraction samples were analyzed for their NO_2_^–^ concentrations using ion chromatography (Dionex IonPac AS22 with a 3.5 mM carbonate and 1.0 mM bicarbonate elution solution) at the Environmental Chemistry facility at Brown University. Standard lab protocols for concentration determination were followed that included calibration to anion standards as well as duplicate, blank, and quality control measurements made every 5 to 7 samples. The relative standard deviations were 2.8% for the quantified NO_2_^–^ concentrations of the quality controls (*n* = 7). Denuder blanks were analyzed and were always below the limits of detection for NO_2_^–^ quantification of 1 μmol L^–1^ (*n* = 3).

The NO_2_^–^ samples were analyzed for their Δ^17^O values as previously described.^52,55^ Briefly, the NO_2_^–^ samples were reduced to nitrous oxide (N_2_O) using 2 mL of a 1:1 mixture of 2 M sodium azide and 50% glacial acetic acid.^56^ After a reaction time of at least 45 min, the samples were neutralized with 6 M NaOH, terminating the reaction. The product N_2_O was then purified and concentrated using an automatic purge and trap system and then thermally decomposed into N_2_ and O_2_ using a gold tube heated to 770 °C.^57^ The products N_2_ and O_2_ are separated via a molecular sieve GC column and then introduced into an Isotope Ratio Mass Spectrometer (IRMS; Delta V Plus) for analysis at *m/z* 32, 33, and 34 to compute Δ^17^O. The samples were calibrated relative to nitrate salts that have internationally recognized Δ^17^O values, including USGS34 (Δ^17^O = −0.3‰) and USGS35 (Δ^17^O = 21.6‰).^58,59^ In line with the “identical treatment” principle, the nitrate salts were reduced to NO_2_^–^ and held in a basic solution (pH > 13) before conversion to N_2_O following previously described methods, which do not impact Δ^17^O values.^52^ The calculated Δ^17^O values were corrected for blank and isobaric influences.^55^ The reduced nitrate reference material had excellent precision with Δ^17^O pooled standard deviations of ±0.2‰ (*n* = 16) and ±0.4‰ (*n* = 17) for USGS34 and USGS35, respectively.

To compare with the Δ^17^O(NO_2_) observations, the average Δ^17^O(NO_2_) by hour for March 2015 from the CMAQ simulation was extracted for the nearest grid cell corresponding to the sample location. This was conducted because the model simulations were unavailable for the sampling dates in March 2018. Thus, we considered a monthly model average to be a better representation of the observations. The model Δ^17^O(NO_2_) simulations correspond to the average March conditions for NO_2_ production by hour, and we evaluated both the average and standard deviations of the model simulations.

### Spatiotemporal Δ^17^O(HNO_3_) in the Northeastern US

The model simulations of spatiotemporal Δ^17^O(HNO_3_) values were compared with recent observations made from the US EPA CASTNET Program that included the following sites: Connecticut Hill, NY (CTH110; 42.40° N, 76.65° W), Abington, CT (ABT147; 41.84° N, 72.01° W), and Woodstock, NH (WST109; 43.94° N, 71.70° W). Samples were collected from 23 December 2016 to 28 December 2018 using a three-stage filter pack, with HNO_3_ collected on Nylon filters following standard laboratory protocols of the CASTNET program.^60^ After collection, the samples were sent to Brown University for Δ^17^O analysis (as well as δ^15^N and δ^18^O). While the model simulation was conducted for 2015 and compared to observations for 2017 and 2018, there were insignificant interannual observed Δ^17^O(HNO_3_) variabilities, and full details on the samples, isotope analysis, and trends can be found in previous reports.^12,61^ At each sample site, the monthly average Δ^17^O was calculated, and the data is reported as the mean ± standard deviation (*x̅* ± 1σ). To compare with these observations, daily Δ^17^O(NO_2_) and Δ^17^O(HNO_3_) were calculated for the nearest grid cell to the observations. The model Δ^17^O(HNO_3_) output corresponds to the 24 h average HNO_3_ production at the considered grid cell.

## Results and Discussion

### Model Δ^17^O Simulations

#### Northeast US Domain Δ^17^O Simulations

**Figure 2:**
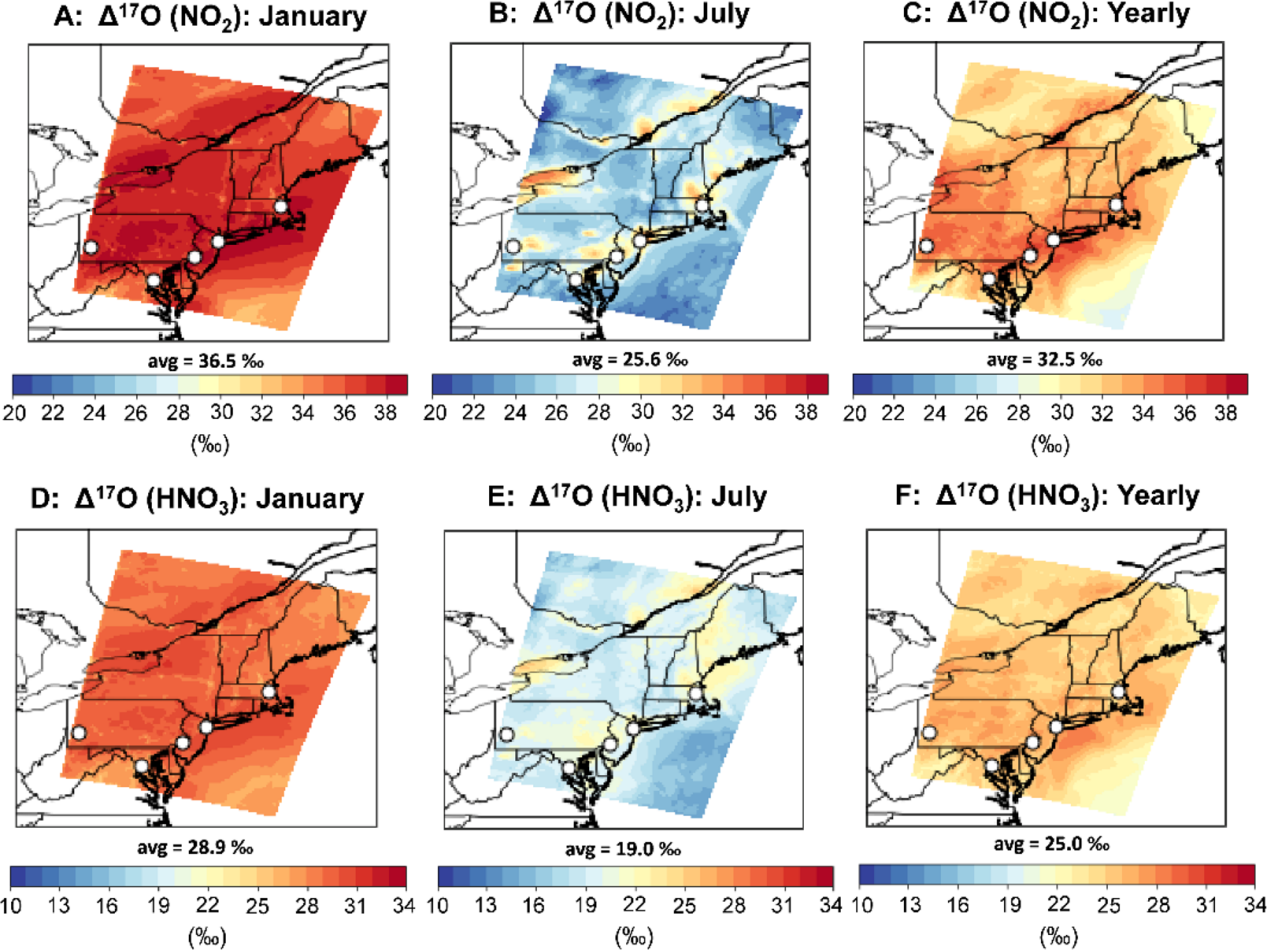
Simulated Δ^17^O(NO_2_) and Δ^17^O(HNO_3_) for the northeastern US for January and July 2015 using the CMAQ with the cb6r3-ae07-NPC mechanism. The Δ^17^O simulations were based on NO_2_ and HNO_3_ production for the lowest 1,000 m of the atmosphere. The locations of major cities in the northeastern US including Boston, MA, New York City, NY, Pittsburgh, PA, Philadelphia, PA, and Washington, DC are indicated by the circles.

The model simulated Δ^17^O(NO_2_) and Δ^17^O(HNO_3_) for the lower 1,000 m of the atmosphere, indicated spatiotemporal differences (Figure 2). This calculation was conducted by summing the total production rate of the various NO_2_ and HNO_3_ formation pathways from the lowest 15 grid boxes and calculating relative production rates via each formation pathway to simulate Δ^17^O for each model simulation day. We considered NO_2_ and HNO_3_ production within the lowest 1,000 m for the domain simulation to be consistent with the previous GEOS-Chem Δ^17^O modeling approach and because most of NO_2_ and HNO_3_ production occurs for the lower atmosphere.^32^ However, considering the lowest 1,000 m may not represent all conditions, such as shallow boundary layer heights. Across the northeastern US domain, the simulated Δ^17^O(NO_2_) and Δ^17^O(HNO_3_) were higher during winter relative to summer, consistent with expectations and previous Δ^17^O simulations.^16,30−32^ For January 2015, Δ^17^O(NO_2_) and Δ^17^O(HNO_3_) averaged (36.5 ± 1.4‰) and (28.9 ± 1.1‰), respectively, across the domain. There were slight spatial differences in Δ^17^O(NO_2_) and Δ^17^O(HNO_3_) during January 2015, in which urban regions tended to have lower values due to increased surface emissions of NO that were not completely photochemically cycled. For July 2015, Δ^17^O(NO_2_) and Δ^17^O(HNO_3_) averaged (25.6 ± 2.3‰) and (19.0 ± 1.9‰), respectively. There were Δ^17^O spatial variabilities, particularly during July, in which Δ^17^O(NO_2_) tended to be higher near urban areas due to elevated O_3_ levels. The higher Δ^17^O(NO_2_) values were proportionally transferred to the urban-produced HNO_3_. Available Δ^17^O observations across urban to rural gradients are quite limited, and future work should investigate these predicted Δ^17^O spatial variabilities.

The fractional contributions of NO_2_ and HNO_3_ formation pathways indicate seasonal changes in NO_*x*_ oxidation chemistry, reflecting a shift from O_3_ to RO_2_/HO_*x*_ chemistry (Figure 2; Table 1). During January, NO_2_ production was dominated by NO_photo_ +O_3_ with a fractional production of 0.916 across the domain (Figure S2). During July, NO_photo_ + O_3_ remained the dominant contributor to NO_2_ production with a fractional production of 0.605 (Figure S3). During the summer, the NO_2_ fractional production via NO_photo_+O_3_ was diminished relative to winter due to the increased concentrations of photochemically produced RO_2_/HO_2_ that played an important role in summertime NO oxidation (Table 1). Across the domain, HNO_3_ production was dominated by N_2_O_5_ hydrolysis during the winter, with a fractional production of 0.589 (Figure S5). During summer, HNO_3_ production from N_2_O_5_ hydrolysis was significantly diminished due to increased temperatures and photolysis, leading to lower N_2_O_5_ concentrations and the increased generation of photochemical oxidants (Figure S6). During summer, HNO_3_ production was dominated by NO_2_ + OH, followed by RONO_2_ hydrolysis (derived from both RO_2_ + NO and R+NO_3_) with fractional productions of 0.581 and 0.270, respectively.

Overall, the annual average Δ^17^O was (32.5 ± 1.8‰) and (25.0 ± 1.4‰) for NO_2_ and HNO_3_, respectively. These relatively high values reflect that, across the domain, the annual NO_2_ production was dominated by reactions involving O_3_, followed by HO_2_/RO_2_ (Table 1). NO_2_ production via reactions involving chlorine monoxide (ClO) was negligible. The annual HNO_3_ production in the northeastern US domain was dominated by reactions involving NO_2_ + OH, followed by N_2_O_5_ + hydrolysis, RONO_2_ (from R + NO_3_) hydrolysis, NO_3_ + VOC, N_2_O_5_ + H_2_O, RONO_2_ (from RO_2_ + NO) hydrolysis, and ClNO_3_ hydrolysis, with near negligible contributions from NO_2_ hydrolysis (Table 1). We note that the simulation of reactive chlorine chemistry has been a challenge for 3D atmospheric chemistry models.^62^ The chemical mechanism we utilized includes reactive chlorine chemistry, which is important in many coastal and inland areas of the US.^38^ Previous studies that have incorporated chlorine chemistry in the mechanisms utilized in CMAQ have indicated its importance for air quality and atmospheric nitrate production in Europe and China.^63,64^ While it is challenging to know how well reactive chlorine chemistry is simulated at our study location, the NO + ClO and ClNO_3_ hydrolysis reactions are high-end Δ^17^O pathways (Table 1).^32^ Thus, if the Δ^17^O model simulations were biased low relative to observations, this could potentially implicate the under prediction of reactive chlorine chemistry in the model chemistry.

#### Simulated Δ^17^O by Altitude

**Figure 3:**
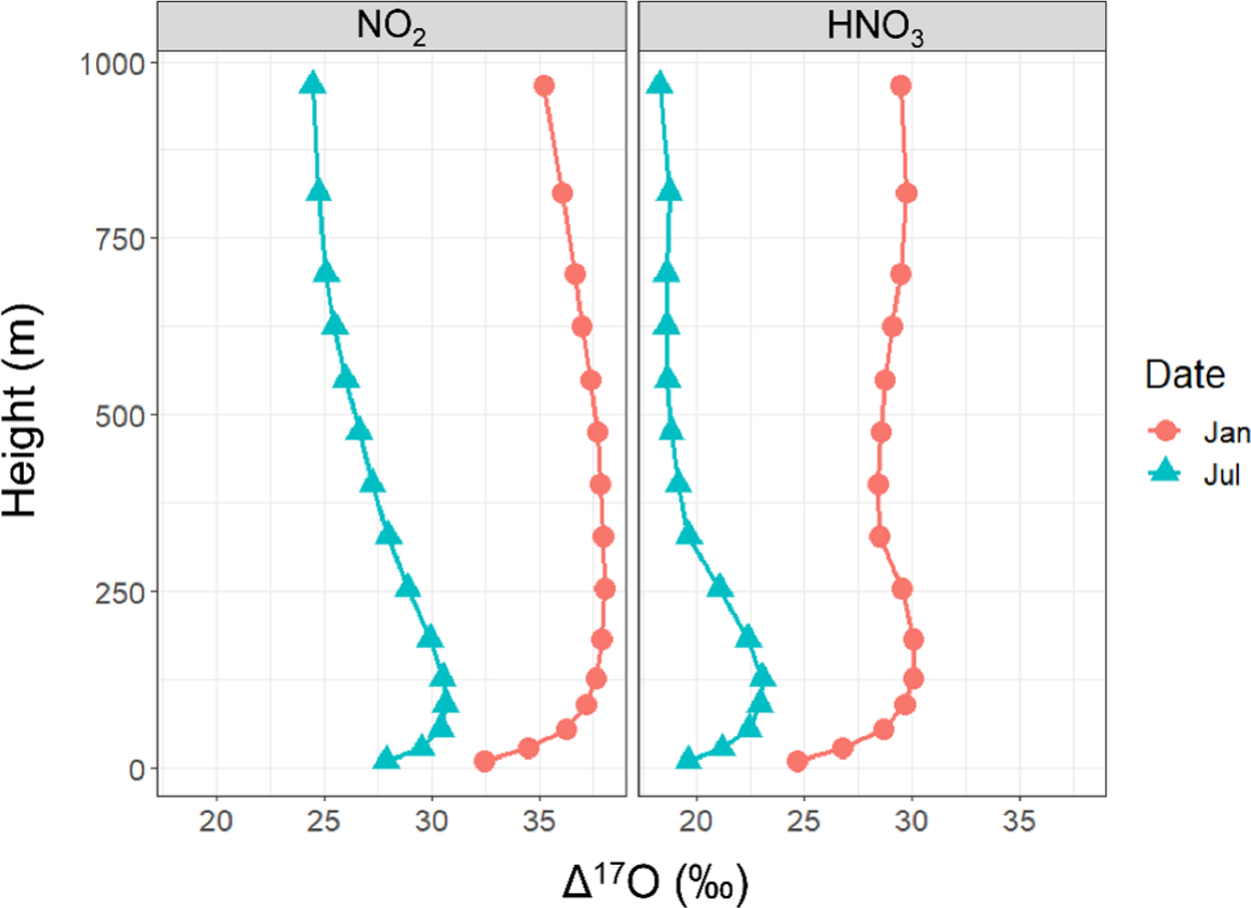
Simulated 24 h average Δ^17^O(NO_2_) and Δ^17^O(HNO_3_) for Providence, RI, US, as a function of atmosphere layer height for January and July 2015.

The model simulations also reveal differences in the modeled Δ^17^O values of NO_2_ and HNO_3_ with altitude (Figure 3). To illustrate this relationship, we separately extracted Δ^17^O simulations for atmospheric layers 1–15 in the CMAQ output files that correspond approximately to the lowest 1,000 m of the atmosphere for Providence, Rhode Island, US (41.82° N, 71.41° W) focusing on representative winter (January 2015) and summer (July 2015) scenarios. The model simulations show that the 24 h average Δ^17^O values of NO_2_ and HNO_3_ were relatively low near the surface and increased gradually with altitude to ∼150 m during the January and July 2015 simulations. The relatively low Δ^17^O values near the surface reflect increased NO emissions and incomplete photochemical cycling that dilutes Δ^17^O(NO_2_) and Δ^17^O(HNO_3_) (Figures S7–S8).

In January 2015, NO_2_ formation was primarily driven by O_3_ oxidation (i.e., NO+O_3_ and NO_photo_+O_3_) across all altitude layers, with an average fractional production of 0.985 ± 0.008 (Figure S7). Thus, the altitude dependence observed in Δ^17^O(NO_2_) in January 2015 reflects the impact of near-surface NO emissions. The relative fraction of non-photochemically cycled NO reacting with O_3_ (i.e., NO+O_3_) peaked at 0.277 for the grid cell closest to the surface. As the altitude increased, the contribution of NO+O_3_ diminished, constituting less than 2% of NO_2_ formation above 200 m. Between 200 and 500 m, the NO_photo_ +O_3_ reaction became the predominant pathway for NO_2_ formation, contributing over 97% of NO_2_ production (Figure S7). Above 500 m, while this pathway remained dominant, there was an increasing relative importance of RO_2_/HO_2_ reactions, leading to slightly lower Δ^17^O(NO_2_) values from 500 to 1,000 m (Figure 3).

Throughout January 2015, the model simulation indicated oscillations in the relative importance of HNO_3_ formation via NO_2_ + OH and N_2_O_5_ heterogeneous reactions (Figure S8). Near the surface, HNO_3_ formation through the NO_2_ + OH pathway was the highest, with a relative fractional production of 0.556. As the altitude increased up to approximately 200 m, the fractional production of HNO_3_ via NO_2_ + OH decreased, while the fractional production via N_2_O_5_ heterogeneous chemistry increased up to 0.657 (Figure S8). Between 200 and 500 m, the relative importance of HNO_3_ production via NO_2_ + OH increased with height, reaching a maximum fractional production of 0.890, balanced by a decrease in HNO_3_ fractional production via N_2_O_5_ heterogeneous reactions. From 500 to 1,000 m, the fractional production of HNO_3_ via NO_2_ + OH decreased, while production via N_2_O_5_ heterogeneous reactions increased with height. The balance between the altitude dependence of Δ^17^O(NO_2_) and changes in the fractional formation pathways of HNO_3_ contributes to the altitude dependence of the simulated Δ^17^O(HNO_3_).

In July 2015, the Δ^17^O(NO_2_) altitude profile resembled that of January 2015 but with lower values due to increased relative formation of NO_2_ via RO_2_/HO_2_ reactions (i.e., NO+RO_2_, NO+HO_2_, NO_photo_+RO_2_, and NO_photo_+HO_2_) that averaged 0.23 ± 0.06 across the considered layer heights. The NO_2_ fractional production via NO + O_3_ was the highest near the surface in July, reaching a maximum of 0.197 for the grid cell closest to the surface. This resulted in the lowest simulated Δ^17^O(NO_2_) values near the surface (Figure 3). As the altitude increased, the fractional production of NO_2_ via NO+O_3_ diminished, stabilizing at an average of 0.071 ± 0.007 from approximately 50 to 1000 m. Above the surface layer, the fractional production of NO_2_ via NO_photo_ + O_3_ increased with height up to approximately 100 m, causing an initial increase in Δ^17^O(NO_2_) with altitude (Figure 3). Above 100 m, the fractional production of NO_2_ via RO_2_/HO_2_ increased, leading to lower Δ^17^O(NO_2_) with height.

Similar to the January 2015 simulation, HNO_3_ formation in July 2015 indicated the oscillating importance of various formation pathways (Figure S8). Near the surface, HNO_3_ formation was dominated by NO_2_ + OH, with a fractional production of 0.845. From the surface to approximately 125 m, the fractional production of HNO_3_ via NO_2_+OH diminished with height, balanced by an increase in the fractional production of HNO_3_ via N_2_O_5_ hydrolysis, reaching a maximum of 0.346. Between approximately 125 and 500 m, the fractional production of HNO_3_ via NO_2_ + OH increased with height, reaching a maximum value of 0.890, while HNO_3_ fractional production via N_2_O_5_ heterogeneous reactions decreased with height. From approximately 500 m to 1,000 m, the relative importance of NO_2_ + OH slightly diminished with height and N_2_O_5_ heterogeneous reactions increased with height. The fractional production of HNO_3_ via RONO_2_ hydrolysis (from RO_2_+NO and R+NO_3_) and NO_3_ + HC increased as a function of altitude, reaching a maximum of 0.0478 and 0.0237 at the highest considered atmosphere layer (Figure S8).

### Comparisons of Model and Observations

#### Urban Δ^17^O(NO_2_) Diel Cycle

**Figure 4:**
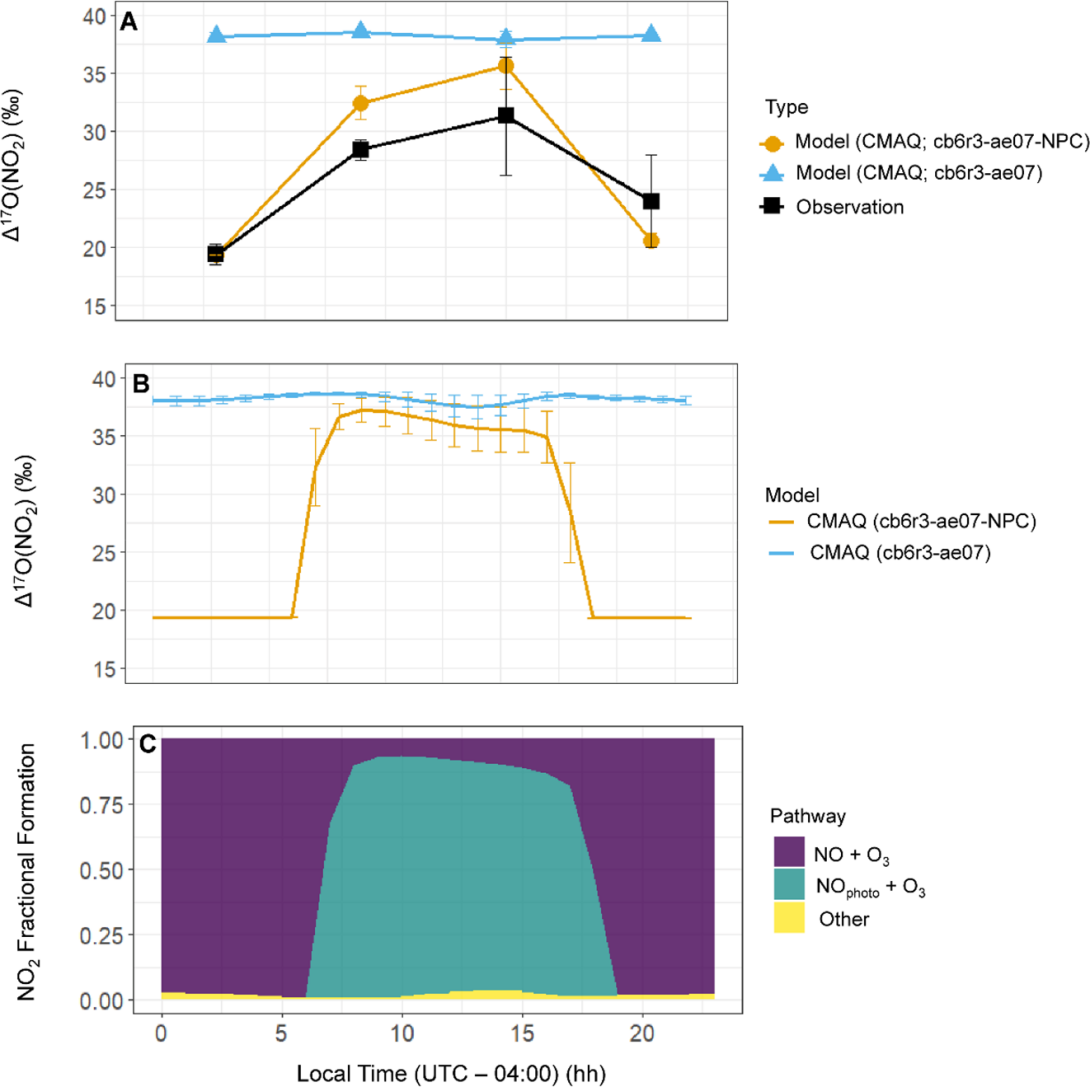
Model and observations of the diel Δ^17^O(NO_2_) in Rumford, RI. (A) The comparison of Δ^17^O(NO_2_) observations (black data points) to the model simulations that were averaged for 6 h and mass-weighted by NO_2_ concentrations to correspond with the Δ^17^O(NO_2_) observations. The data points represent the average values, and the *y*-axis error bars correspond to the standard deviation of the samples and model simulations. (B) The comparison between diel Δ^17^O(NO_2_) simulations using the CMAQ model with the newly developed NO_*x*_ photochemical tagging approach (cb6r3-ae07-NPC) and the traditional approach (cb6r3-ae07) assuming all NO is photochemically cycled for March 2015 averaged by hour. (C) The model diel fractional formation pathways of NO_2_ based on the cb6r3-ae07-NPC are also indicated including NO + O_3_, NO_photo_ + O_3_, and all others (NO + RO_2_, NO + HO_2_, NO + ClO, NO_photo_ + RO_2_, NO_photo_ + HO_2_, NO_photo_ + ClO). All model simulations correspond to the lowest 100 m of the atmosphere.

The observations of Δ^17^O(NO_2_) in Rumford, RI, during March 2018 revealed a prominent diel cycle with the highest values observed during daytime hours, while the lowest values were observed at night (Figure 4A). These findings also closely align with prior ambient Δ^17^O(NO_2_) measurements conducted in Grenoble, France.^20^ During the early morning sampling period, spanning from 0:00 to 6:00 am, Δ^17^O(NO_2_) reached its minimum, with an average of (19.4 ± 0.9‰; *n* = 3). These low Δ^17^O(NO_2_) values stem from nighttime emissions of NO near the surface that are subsequently oxidized by O_3_. Employing an oxygen-isotope mass balance and assuming Δ^17^O(NO_source_) = 0‰ and Δ^17^O(O_3_^term^) = 39‰, a calculated nighttime Δ^17^O(NO_2_) value of 19.5‰ closely matches the observed values. With the onset of daylight, Δ^17^O(NO_2_) values increased to (28.4 ± 0.9‰; *n* = 3) and (31.4 ± 5.1‰; *n* = 3) for collections conducted between 06:00–12:00 and 12:00–18:00, respectively. This increase in Δ^17^O(NO_2_) is attributed to NO_*x*_ photochemical cycling, in which Δ^17^O(NO_*x*_) achieves isotope equilibrium with the oxidants involved in the NO_*x*_ cycle.^49^ Post-sunset, Δ^17^O(NO_2_) decreased, averaging (24.0 ± 4.0‰; *n* = 3). This reduction in Δ^17^O(NO_2_) relative to daytime levels can be attributed to new nocturnal NO emissions that are oxidized to NO_2_, which subsequently mixes with the daytime photochemically cycled NO_2_ with a higher Δ^17^O(NO_2_) value. As the night progresses, Δ^17^O(NO_2_) slowly converges towards the nighttime Δ^17^O(NO_2_) production value of approximately 19.5‰. The balance between the nocturnal production of NO_2_ with a lower Δ^17^O(NO_2_) value, mixing with photochemically cycled NO_2_ possessing a higher Δ^17^O(NO_2_) value, results in the post-sunset collection period exhibiting a higher Δ^17^O(NO_2_) compared to the early morning period before sunrise.

CMAQ was utilized to simulate the diel Δ^17^O(NO_2_) values to directly compare to the near-surface observations (Figure 4B). We note that it can be challenging to directly compare our near-surface Δ^17^O(NO_2_) observations to the model simulated values since the employed implicit tagging mechanism tracks the instantaneous NO_2_ production by its various reaction pathways within each grid cell without the explicit incorporation of horizontal or vertical transport. Due to the relatively short lifetime of NO_2_ on the order of several hours, it may not mix throughout the boundary layer. Thus, diel simulations Δ^17^O(NO_2_) were conducted for each atmosphere layer grid cell from the surface up to ∼1,000 m binned by hour (Figure S9). These simulations indicated lower Δ^17^O(NO_2_) values near the surface due to increased NO emissions that do not completely photochemically cycle before oxidation to NO_2_, as well as lower Δ^17^O(NO_2_) values above approximately 500 m due to increased importance of HO_2_/RO_2_ chemistry (Figure S10), consistent with our previous layer height Δ^17^O(NO_2_) discussion (Figure 3). We also considered Δ^17^O(NO_2_) for various altitude bins from the surface to ∼100, ∼250, ∼500, and ∼1,000 m (Figure S11). Overall, these considered altitude bins indicate minor differences in the simulated diel Δ^17^O(NO_2_) that differed no more than 0.4% in their 24 h non-weighted averages (Figure S11). This is due to the vast majority of NO_2_ production occurring near the surface (Figure S12). Consequently, in our assessment of the near-surface Δ^17^O(NO_2_) observations compared to the model simulations, we will utilize the near-surface altitude bin (surface to 100 m). We anticipate that this selection will not notable influence the Δ^17^O(NO_2_) comparison.

Overall, the diel Δ^17^O(NO_2_) simulation with 1 h bins averaged over March 2015 utilizing the newly developed cb6r3-ae07-NPC mechanism indicates a substantial diurnal pattern with the lowest values during the nighttime and the highest values during the daytime (Figure 4B). This simulation suggests that, in the absence of NO_2_ photolysis, Δ^17^O(NO_2_) stabilizes around 19.5‰, due to fresh NO emissions that are oxidized to NO_2_ via reaction with O_3_ (Figure 4C). During the daytime, Δ^17^O(NO_2_) experiences an increase due to photochemical cycling, with peak values occurring around sunrise and sunset. Simulated midday values of Δ^17^O(NO_2_) are slightly lower, attributed to elevated RO_2_/HO_2_ production. In comparison, we also calculated Δ^17^O(NO_2_) using the traditional Δ^17^O modeling approach, which assumes all NO is photochemically cycled (Figure 4B). This simulation indicates that Δ^17^O(NO_2_) is relatively steady throughout the day with an average of 38.2 ± 0.3‰, with slightly lower values midday due to increased photochemical production of RO_2_/HO_2_ production (Figure 4A). The Δ^17^O(NO_2_) diurnal observations most closely align with the newly developed cb6r3-ae07-NPC mechanism (Figure 4A), in which the lowest Δ^17^O(NO_2_) values are observed during the nighttime and highest values are observed during the daytime.

For a direct comparison to the near-surface Δ^17^O(NO_2_) observations, the model output was mass-weighted based on the modeled NO_2_ concentrations (Figure S13) for the specified 6 h NO_2_ collection windows to be consistent with the observations (Figure 4A). In general, the Δ^17^O(NO_2_) simulation using the newly developed cb6r3-ae07-NPC mechanism closely aligns with observations and diurnal patterns, even considering the relatively coarse temporal resolution of available measurements. The model effectively predicts Δ^17^O(NO_2_) observed during the early hours before sunrise, underscoring the significance of nocturnal NO emissions that contribute to the dilution of photochemically cycled Δ^17^O(NO_2_) values. Furthermore, the model accurately captures the trend of an increase in Δ^17^O(NO_2_) as NO_2_ in the atmosphere undergoes photochemical cycling^49^ We note that the Δ^17^O(NO_2_) observations tend to be slightly lower than the Δ^17^O(NO_2_) simulations during the daytime (Figure 4B). This discrepancy may be due to the impact of near-surface NO emissions at the observation site, which included a nearby parking lot that may not have been accurately captured in the model given its spatial resolution. Still, the model with the cb6r3-ae07-NPC mechanism captured the observed Δ^17^O(NO_2_) diel trend and was a significant improvement compared to previous Δ^17^O model frameworks (Figure 4). Further, a slight deviation between the Δ^17^O(NO_2_) model and observations is noticeable during the early nighttime period, where observations suggest a gradual Δ^17^O(NO_2_) decline post-sunset due to the mixing of photochemically cycled Δ^17^O(NO_2_) and newly produced NO_2_ derived from nocturnal NO emissions. In contrast, the simulated Δ^17^O(NO_2_) only considers the new production of NO_2_, thus simulating a lower Δ^17^O(NO_2_) value during the early nighttime period. Despite these minor discrepancies, the 24 h non-weighted average Δ^17^O(NO_2_) between the simulated value using CMAQ with the cb6r3-ae07-NPC mechanism and observations closely agree, yielding averages of (27.3 ± 8.1‰; *n* = 24) and (25.8 ± 5.6‰; *n* = 12), respectively. Therefore, the slight errors in Δ^17^O(NO_2_) simulations should not exert a substantial influence when comparing 24 h averaged observations or when considering HNO_3_ production over a 24 h period.

#### Spatiotemporal Δ^17^O(HNO_3_) in the Northeastern US

**Figure 5:**
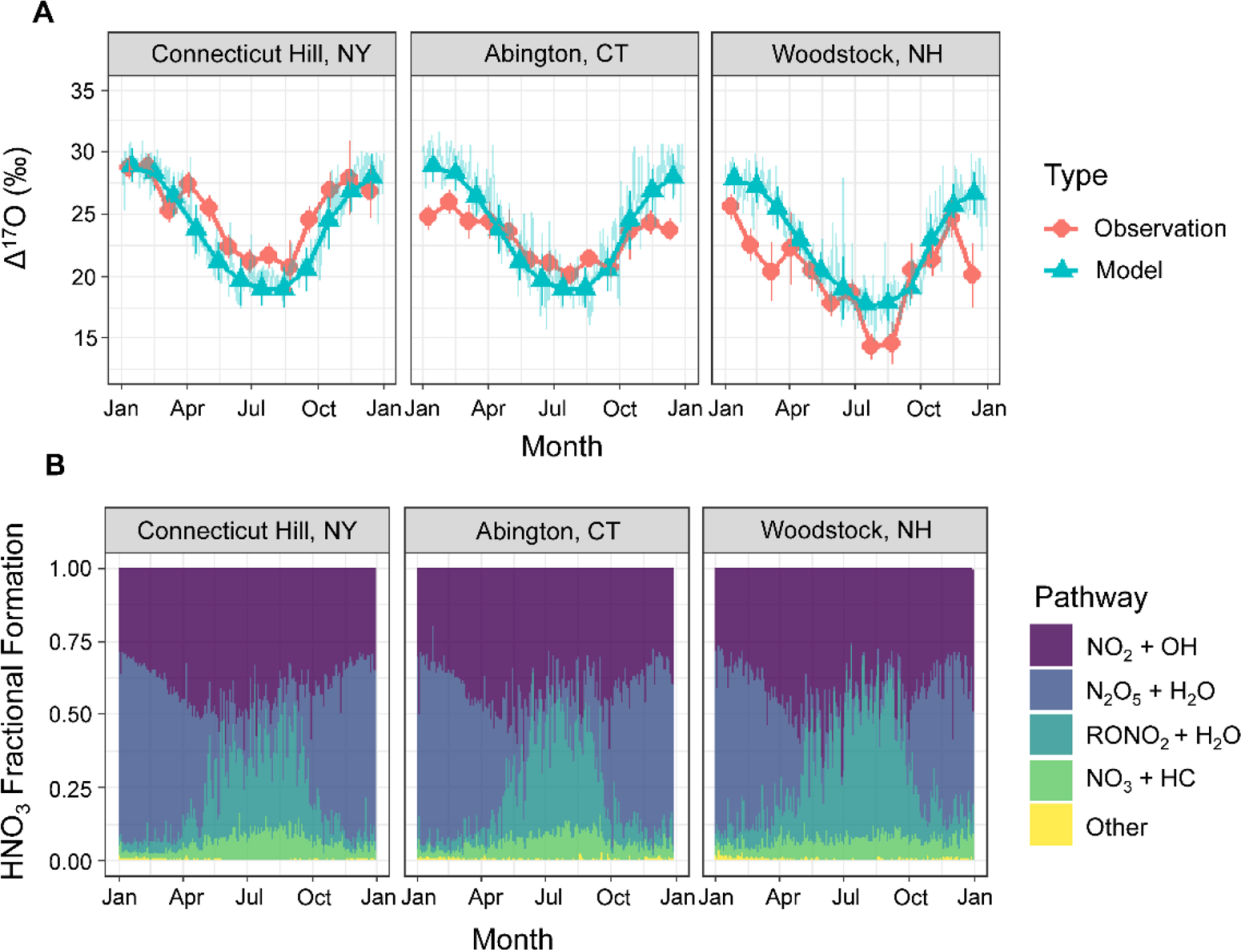
(A) Comparison between the Δ^17^O(HNO_3_) observations from US EPA CASTNET sites and the simulated values using the CMAQ model with the cb643-ae07-NPC mechanism in the northeastern US. The Δ^17^O(HNO_3_) observations correspond to approximate monthly averaged values. The model Δ^17^O(HNO_3_) simulation includes the daily values (light lines) and the monthly averaged values (dark lines and data points). The error bars correspond to the standard deviations of the observations and model simulation. (B) The model simulated HNO_3_ fractional formation pathways at the considered CASTNET sites, including NO_2_ + OH, N_2_O_5_ reactions (both gas phase and heterogenous), RONO_2_ (from RO_2_+NO and R+NO_3_) hydrolysis, NO_3_ + HC, and others (NO_2_ hydrolysis and chlorine nitrate hydrolysis). The CMAQ model simulation was conducted for the northeastern US domain for 2015 and considered the lowest 1,000 m of the atmosphere. The Δ^17^O(HNO_3_) observations include samples collected from 2017–2018 as previously reported.^12^

The model simulations of Δ^17^O(HNO_3_) for 2015 were compared with monthly average Δ^17^O(HNO_3_) observations for three CASTNET sites in the northeastern US for 2017–2018 (Figure 5). These model simulations considered the Δ^17^O(HNO_3_) values derived from the grid cell nearest to the observation sites and for HNO_3_ production occurring below an approximate altitude of 1,000 meters. The Δ^17^O(HNO_3_) observations represent the average of 2017–2018 grouped by month. The samples were pooled by month to ensure enough sample material for Δ^17^O(HNO_3_) analysis as previously described.^12^

The Δ^17^O(HNO_3_) observations for the considered US EPA CASTNET sites in the northeastern US indicated a discernible and statistically significant seasonal pattern at all three monitoring sites, characterized by starkly higher values during the winter months in contrast to the summer season due to the seasonal shift from O_3_ to RO_2_/HO_*x*_ chemistry, as previously reported.^12^ Impressively, the model simulations replicated these observed seasonal fluctuations in both local and regional Δ^17^O(HNO_3_) values. The model predicted that HNO_3_ formation was dominated by the N_2_O_5_ hydrolysis during the winter, representing (59.2 ± 15.7%) of HNO_3_ production at the considered CASTNET sites during January. During the summer, HNO_3_ formation was dominated by NO_2_ + OH and RONO_2_ (from RO_2_+NO and R+NO_3_) hydrolysis that represented (48.5 ± 13.1%) and (34.6 ± 12.5%) of HNO_3_ production, respectively, during July at the CASTNET sites. This finding highlights the importance of improving our understanding of organic nitrate chemistry as a pathway for HNO_3_ formation as anthropogenic NO_*x*_ emissions decrease, and the importance of organic nitrate chemistry may increase, as indicated from GEOS-Chem simulations in the US.^32^

Additionally, the Δ^17^O(HNO_3_) observations indicated statistically significant spatial differences (*p* < 0.01) among the monitoring sites, with the Δ^17^O(HNO_3_) values increasing in the following order: WST109 (20.2 ± 3.6‰; *n* = 26); ABT147 (23.1 ± 2.1‰; *n* = 27); and CTH110 (25.0 ± 3.1‰; *n* = 25). Correspondingly, the CMAQ model simulations also yielded a statistically significant spatial gradient (*p* < 0.01), representing a noteworthy advancement relative to prior GEOS-Chem simulations, which had failed to capture these Δ^17^O(HNO_3_) distinctions at these specific sites.^12^ The annual CMAQ simulated Δ^17^O(HNO_3_) values increased in the order of WST109 (22.7 ± 4.0‰; *n* = 365); CTH110 (24.2 ± 3.8‰; *n* = 365); and ABT147 (24.5 ± 4.1‰; *n* = 365). The model predicted spatial differences in Δ^17^O(HNO_3_) were due to differences in simulated HNO_3_ production (Figure 5). Across the three sites, the annual modeled production of HNO_3_ was primarily influenced by three key reactions: NO_2_ + OH, N_2_O_5_ hydrolysis, and RONO_2_ (from RO_2_+NO and R+NO_3_) hydrolysis. These reactions exhibited varying seasonality and relative significance at each site. The model simulated annual HNO_3_ production demonstrated similar patterns at the CTH110 and AB147 sites, with fractional contributions from NO_2_ + OH, N_2_O_5_ hydrolysis, and RONO_2_ hydrolysis accounting for 49.4%, 30.9%, and 11.7% at CTH110 and 49.3%, 30.4%, and 11.8% at ABT147. In contrast, HNO_3_ production at the WST109 site, the most rural and with the lowest NO_*x*_ emission density near the considered CASTNET sites (Figure 1), displayed a distinct profile characterized by higher contributions from RONO_2_ hydrolysis and lower contributions from N_2_O_5_ hydrolysis compared to the other CASTNET sites. The annual fractional production of HNO_3_ at WST109 was 49.4%, 24.1%, and 18.7% for NO_2_ + OH, N_2_O_5_ hydrolysis, and RONO_2_ hydrolysis, respectively.

The model’s accurate representation of the relatively lower Δ^17^O(HNO_3_) observations at the WST109 site, relative to the other monitoring sites, is attributed to the heightened significance of RO_2_ chemistry, which includes the enhanced formation of HNO_3_ through RONO_2_ hydrolysis (Figure 5). However, it is noteworthy that the model faced challenges in accurately replicating the subtle spatial Δ^17^O(HNO_3_) distinctions observed between CTH110 and ABT147. In particular, the model tended to simulate Δ^17^O(HNO_3_) high relative to the observations at ABT147. This disparity indicates an over-incorporation of O_3_ in the HNO_3_ product, which could indicate that the N_2_O_5_ hydrolysis reaction, a high Δ^17^O(HNO_3_)-endmember and important reaction during winter, was overpredicted as a pathway for HNO_3_ formation at this site. Additionally, it is important to point out that the model difference relative to observations could be due to the transport of regionally produced HNO_3_ to the monitoring sites. The Δ^17^O(HNO_3_) simulations consider local HNO_3_ production only and do not consider the influence of transport. Even with this simplification, the computed Δ^17^O(HNO_3_) model averages closely mirrored the observed average values. To assess the model’s performance, the root mean square error (RMSE) between the monthly averaged Δ^17^O(HNO_3_) observations and their corresponding local Δ^17^O(HNO_3_) simulated values were calculated, resulting in values of 1.9‰, 2.7‰, and 3.2‰ for CTH110, ABT147, and WST109, respectively. The overall RMSE for the model comparison to all observations was 2.6‰. The RMSEs are near the Δ^17^O measurement precision of 1–2‰, as previously reported.^12^ These calculated errors underscore the model’s capability to capture the intricate Δ^17^O(HNO_3_) variabilities exhibited across the sites. Expansion of Δ^17^O(HNO_3_) observations and comparison to CMAQ expectations will help enable adjustment/corrections to model chemistry.

## Implications

This work presents a new model framework for evaluating atmospheric NO_*x*_ photochemical cycling and HNO_3_ formation using oxygen isotopes. This new tool has been incorporated into the US EPA CMAQ model, representing the second 3D atmospheric chemistry model with this capability. A new modeling framework was developed to accurately simulate the Δ^17^O values of NO_*x*_ photochemical cycling. The preliminary model evaluation demonstrates its ability to accurately simulate the diurnal profile of NO_2_ and spatiotemporal HNO_3_ production. The mechanism indicates that, across the northeastern US domain, approximately 8% of the formed NO_2_ was from NO that had not achieved oxygen isotope photochemical equilibrium, which has important implications for accurately modeling Δ^17^O of NO_2_ and HNO_3_. Overall, the model indicates that annual HNO_3_ formation in the northeastern US is dominated by NO_2_ + OH (46%), N_2_O_5_ hydrolysis (34%), and organic nitrate hydrolysis (12%). The elevated levels of HNO_3_ formed via organic nitrate for this region are intriguing and highlight the continued need to improve our understanding of organic nitrate chemistry. This pathway is expected to increase as anthropogenic NO_*x*_ emissions decrease. Further, the model predicts wide spatiotemporal variation in atmospheric nitrate formation across the northeastern US and interesting Δ^17^O differences across various atmospheric layer heights. Future work will expand the modeling domain across the contiguous US, which will be a key tool for evaluating CMAQ representation of NO_*x*_ chemistry.

The presented model framework is the first 3D chemical transport model that has been shown to be able to capture the diurnal Δ^17^O variability of NO_2_, which is a key step for accurately simulating Δ^17^O of HNO_3_ via explicitly accounting for non-photochemically cycled NO using the generated cb6r3-ae07-NPC mechanism. This approach is a dramatic step forward in our ability to accurately model Δ^17^O values of NO_2_ and HNO_3_. Previous methods for simulating Δ^17^O have assumed that all of the formed NO_2_ derives from photochemically cycled NO, such that Δ^17^O(NO_2_) directly reflects the oxidants involved in NO_*x*_ cycling due to photochemical equilibrium. However, this results in a drastic oversimplification for regions with significant nighttime NO emissions, such as the polluted mid-latitudes. Our new approach more accurately simulates Δ^17^O of NO_2_ and HNO_3_, particularly for regions relevant to air quality and nitrogen deposition-related studies. Further, the model simulations, which consider Δ^17^O values across different atmospheric layers, underscore the unique potential of monitoring the production of NO_2_ and HNO_3_ as a function of atmospheric height and its involvement in NO_*x*_ chemistry. These findings have important implications for future studies on air quality and atmospheric chemistry, as Δ^17^O values could be used to evaluate unique HNO_3_ production pathways near the surface versus aloft. We envision that a comprehensive comparison of Δ^17^O model expectations with observations will significantly aid our understanding of tropospheric oxidation chemistry, with important implications for air quality and deposition studies.

## Supporting Information Available

Summary of the cb6r3-ae07-NPC mechanism, annual 2015 NO_2_ fractional production, January 2015 NO_2_ fractional production, July 2015 NO_2_ fractional production, annual HNO_3_ fractional production, January 2015 HNO_3_ fractional production, July 2015 HNO_3_ fractional production, NO_2_ fractional production as a function of layer height, HNO_3_ fractional production as a function of layer height, Diel NO_2_ fractional production as a function of layer height, Diel Δ^17^O(NO_2_) simulations for various altitude bins, cumulative NO_2_ production as a function of layer height, and averaged modeled NO_2_ mixing ratios for Rumford, RI in March 2015 (PDF)
