# Supplementary material for: Modeling the Oxygen Isotope Anomaly (Δ17O) of Reactive Nitrogen in the Community Multiscale Air Quality Model: Insights into Nitrogen Oxide Chemistry in the Northeastern United States
Source: ACS EST Air. 2024 Apr 22;1(6):451–63. doi: 10.1021/acsestair.3c00056 (PMC11151734; doi:10.1021/acsestair.3c00056)
Supplement: Supplementary file 1 — ea3c00056_si_001.pdf [file ea3c00056_si_001.pdf]

Supplement for:

Modeling the Oxygen Isotope Anomaly ($\Delta^{17}O$) of Reactive Nitrogen in the Community Multiscale Air Quality (CMAQ) Model: Insights into Nitrogen Oxide Chemistry in the Northeastern United States

Wendell W. Walters[1,2*]; Havala, O. T. Pye[2]; Heejeong Kim[3,4]; Meredith G. Hastings[3,4]

[1]Department of Chemistry and Biochemistry, University of South Carolina, Columbia, SC, USA 29208
[2]Office of Research and Development, U.S. Environmental Protection Agency, Durham, NC, USA, 27703
[3]Department of Earth, Environment, and Planetary Sciences, Brown University, Providence, RI 02912
[4]Instiute at Brown for Environment and Society, Brown University, Providence, RI 02912
*Corresponding Author: wendellw@mailbox.sc.edu

Table of Contents

Table S1. Summary of the cb6r3-ae07-NPC mechanism S1-S2

Fig. S1. Annual 2015 NO_2 fractional production S3

Fig. S2. January 2015 NO_2 fractional production....................S4

Fig. S3. July 2015 NO_2 fractional production S5

Fig. S4. Annual HNO_3 fractional production S6

Fig. S5. January 2015 HNO_3 fractional production....................S7

Fig. S6. July 2015 HNO_3 fractional production S8

Fig. S7. NO_2 fractional production as a function of layer height S9

Fig. S8. HNO_3 fractional production as a function of layer height S10

Fig. S9. Diel $\Delta^{17}O(NO_2)$ simulations as a function of layer height....................S11

Fig. S10. Diel NO_2 fractional production as a function of layer height S12

Fig. S11. Diel $\Delta^{17}O(NO_2)$ simulations for various altitude bins S13

Fig. S12. Cumulative NO_2 production as a function of layer height....................S14

Fig. S13. Average modeled NO_2 mixing ratios for Rumford, RI in March 2015 ....S15

Table S1. Summary of the cb6r3-ae07-NPC chemical mechanism to track NO photochemical cycling, including renamed reaction and replicate reactions.

Rxn	Description	Reaction	Replicate
R1	Rename	$NO_2 \rightarrow NO_{photo} + O$	NA
R3	Replicate	$O_3 + NO \rightarrow NO_2$	$O_3 + NO_{photo} \rightarrow NO_2$
R4	Replicate	$O + NO + M \rightarrow NO_2 + M$	$O + NO_{photo} + M \rightarrow NO_2 + M$
R24	Replicate	$NO + NO + O_2 \rightarrow 2.0NO_2$	$NO_{photo} + NO + O_2 \rightarrow 2.0NO_2$<br><br>$NO_{photo} + NO_{photo} + O_2 \rightarrow 2.0NO_2$
R25	Replicate	$HO_2 + NO \rightarrow OH + NO_2$	$HO_2 + NO_{photo} \rightarrow OH + NO_2$
R29	Replicate	$NO_3 + NO \rightarrow 2.0NO_2$	$NO_3 + NO_{photo} \rightarrow 2.0NO_2$
R40	Replicate	$NO + OH \rightarrow HONO$	$NO_{photo} + OH \rightarrow HONO$
R41	Replicate	$NO + NO_2 + H_2O \rightarrow 2.0HONO$	$NO_{photo} + NO_2 + H_2O \rightarrow 2.0HONO$
R53	Replicate	$C_2O_3 + NO \rightarrow NO_2 + MEO_2 + RO_2$	$C_2O_3 + NO_{photo} \rightarrow NO_2 + MEO_2 + RO_2$
R61	Replicate	$CXO_3 + NO \rightarrow NO_2 + ALD2 + XO_2H + RO_2$	$CXO_3 + NO_{photo} \rightarrow NO_2 + ALD_2 + XO_2H + RO_2$
R68	Replicate	$RO_2 + NO \rightarrow NO$	$RO_2 + NO_{photo} \rightarrow NO$
R71	Replicate	$MEO_2 + NO \rightarrow FORM + HO_2 + NO_2$	$MEO_2 + NO_{photo} \rightarrow FORM + HO_2 + NO_2$
R75	Replicate	$XO_2H + NO \rightarrow NO_2 + HO_2$	$XO_2H + NO_{photo} \rightarrow NO_2 + HO_2$
R79	Replicate	$XO_2 + NO \rightarrow NO_2$	$XO_2 + NO_{photo} \rightarrow NO_2$
R83	Replicate	$XO_2N + NO \rightarrow 0.5NTR1 + 0.5NTR2$	$XO_2N + NO_{photo} \rightarrow 0.5NTR1 + 0.5NTR2$
R103	Replicate	$HCO_3 + NO \rightarrow FACD + NO_2 + HO_2$	$HCO_3 + NO_{photo} \rightarrow FACD + NO_2 + HO_2$
R151	Replicate	$ISO_2 + NO \rightarrow 0.1INTR + 0.9NO_2 + 0.673FORM + 0.9ISPD + 0.818HO_2 + 0.082XO_2H + 0.082RO$	$ISO_2 + NO_{photo} \rightarrow 0.1INTR + 0.9NO_2 + 0.673FORM + 0.9ISPD + 0.818HO_2 + 0.082XO_2H + 0.082RO_2$
R167	Replicate	$EPX_2 + NO \rightarrow 0.275GLYD + 0.275GLY + 0.275MGLY + 0.125OH + 0.825HO_2 + 0.375FORM + NO_2 + 0.251CO + 2.175PAR$	$EPX_2 + NO_{photo} \rightarrow 0.275GLYD + 0.275GLY + 0.275MGLY + 0.125OH + 0.825HO_2 + 0.375FORM + NO_2 + 0.251CO + 2.175PAR$
R176	Replicate	$BZO_2 + NO \rightarrow 0.918NO_2 + 0.082NTR2 + 0.918GLY + 0.918OPEN + 0.918HO_2$	$BZO_2 + NO_{photo} \rightarrow 0.918NO_2 + 0.082NTR_2 + 0.918GLY + 0.918OPEN + 0.918HO_2$
R181	Replicate	$TO_2 + NO \rightarrow 0.86NO_2 + 0.14NTR2 + 0.417GLY + 0.443MGLY + 0.66OPEN + 0.2XOPN + 0.86HO_2$	$TO_2 + NO_{photo} \rightarrow 0.86NO_2 + 0.14NTR2 + 0.417GLY + 0.443MGLY + 0.66OPEN + 0.2XOPN + 0.86HO_2$
R207	Replicate	$OPO_3 + NO \rightarrow NO_2 + 0.5GLY + 0.5CO + 0.8HO_2 + 0.2CXO_3$	$OPO3 + NO_{photo} \rightarrow NO_2 + 0.5GLY + 0.5CO + 0.8HO_2 + 0.2CXO_3$
CL5	Replicate	$ClO + NO \rightarrow Cl + NO_2$	$ClO + NO_{photo} \rightarrow Cl + NO_2$
SA01a	Replicate	$TOLRO_2 + NO \rightarrow NO + 0.016SVAVB2 + 0.051SVAVB3 + 0.047SVAVB4$	$TOLRO_2 + NO_{photo} \rightarrow NO +0.016SVAVB2 +0.051SVAVB3 +0.047SVAVB4$

SA03a	Replicate	$XYLRO_2$ + NO → NO + 0.015SVAVB2 + 0.023SVAVB3 + 0.06SVAVB4	$XYLRO_2$ + NO_{photo} → NO + 0.015SVAVB2 + 0.023SVAVB3 +0.06SVAVB4
SA06a	Replicate	$BENZRO_2$ + NO → NO + 0.034SVAVB2 + 0.392SVAVB4	$BENZRO_2$+ NO_{photo} → NO + 0.034SVAVB2 +0.392SVAVB4
SA119	Replicate	$PAHRO_2$ + NO → NO + 0.028SVAVB2 + 0.225SVAVB3 + 0.191SVAVB4	$PAHRO_2$ + NO_{photo} → NO + 0.028SVAVB2 + 0.22SVAVB3 + 0.191SVAVB4
BL18aa	Replicate	$TERPNRO_2$ + NO → NO + 0.688$MTNO_3$	$TERPNRO_2$ + NO_{photo} → NO + 0.688$MTNO_3$

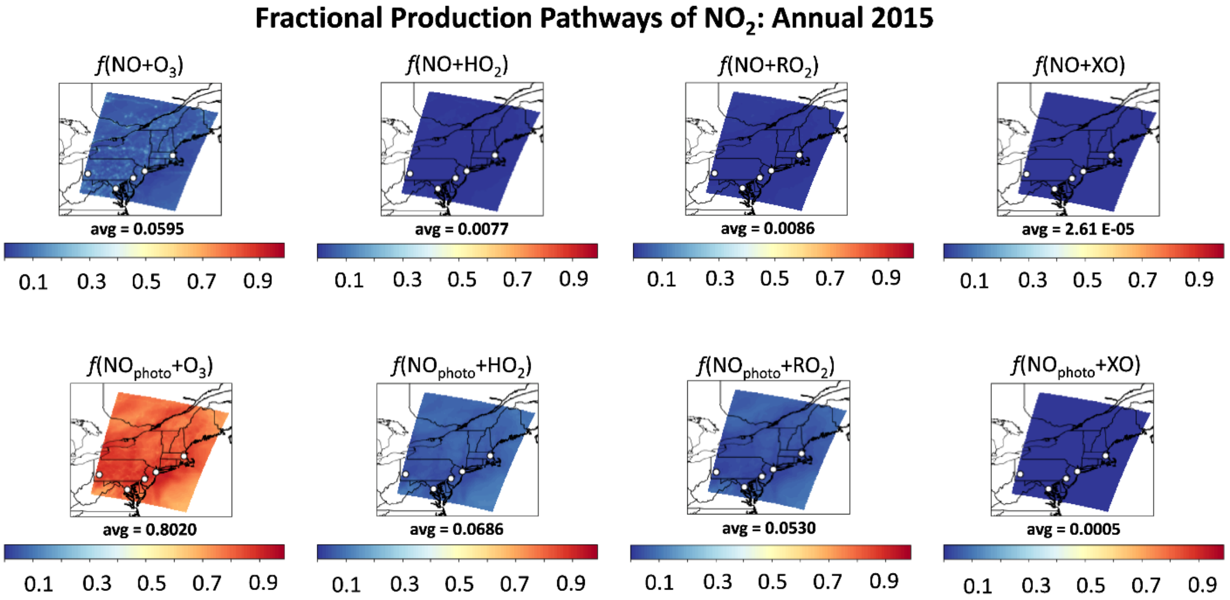

Fig. S1. The CMAQ model simulation of NO_2 fractional production pathways for 2015 in the northeastern US domain for the bottom 1,000 m of the atmosphere. The open data points represent major urban areas, including (from north to south) Boston, MA, New York City, NY, Pittsburgh, PA, Philadelphia, PA, and Washington DC.

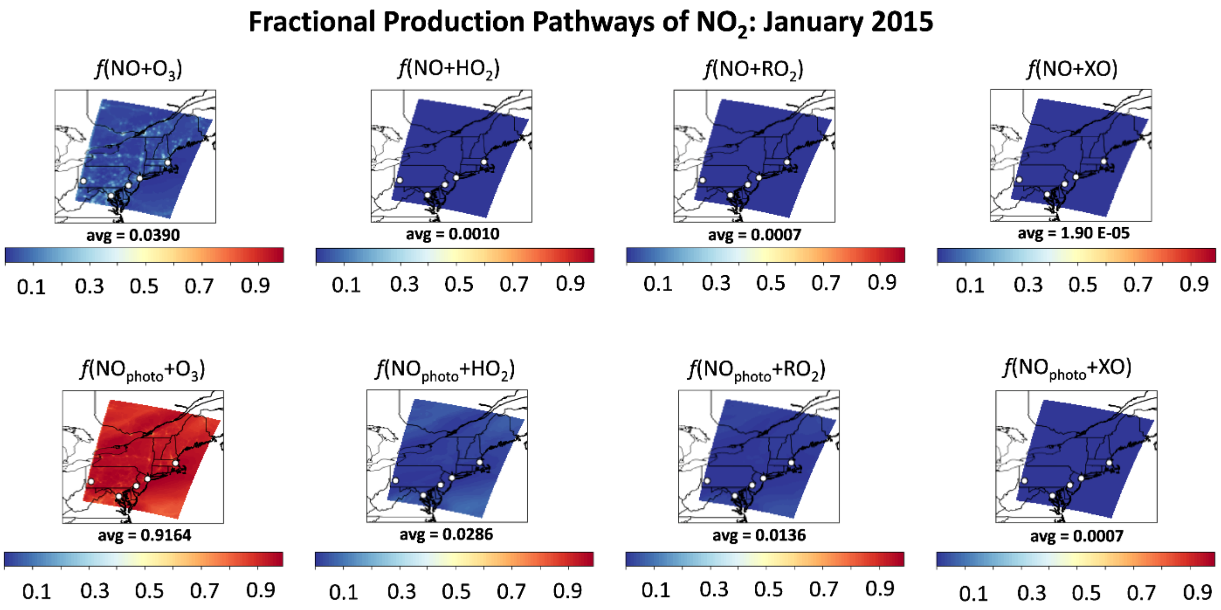

Fig. S2. The CMAQ model simulation of NO_2 fractional production pathways for January 2015 in the northeastern US domain for the bottom 1,000 m of the atmosphere. The open data points represent major urban areas, including (from north to south) Boston, MA, New York City, NY, Pittsburgh, PA, Philadelphia, PA, and Washington DC.

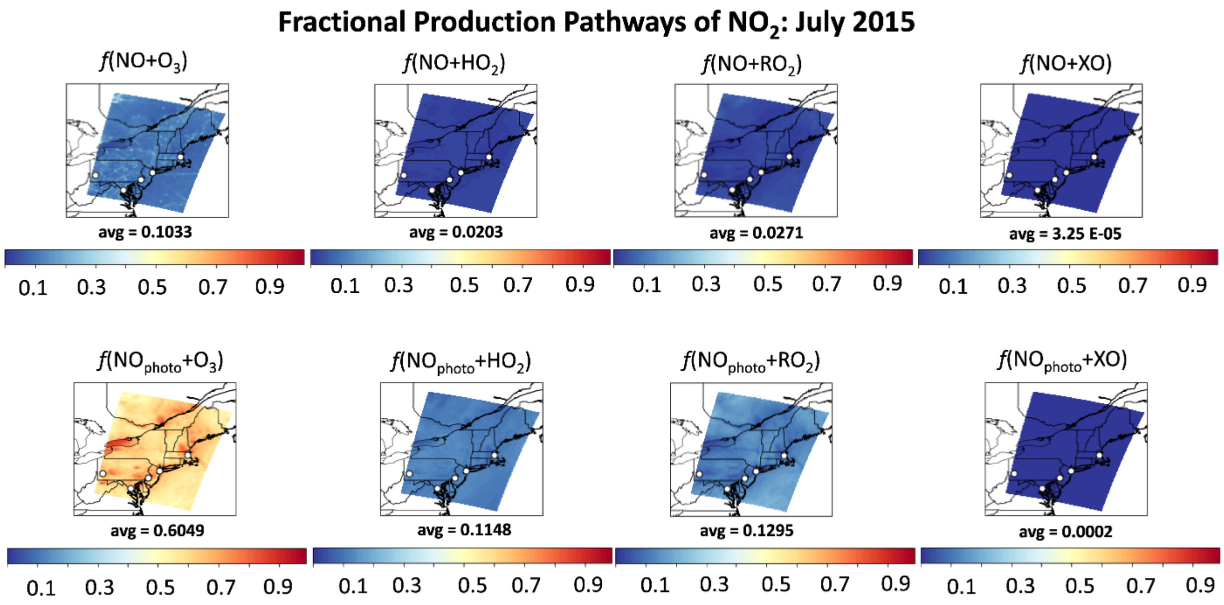

Fig. S3. The CMAQ model simulation of NO_2 fractional production pathways for July 2015 in the northeastern US domain for the bottom 1,000 m of the atmosphere. The open data points represent major urban areas, including (from north to south) Boston, MA, New York City, NY, Pittsburgh, PA, Philadelphia, PA, and Washington DC.

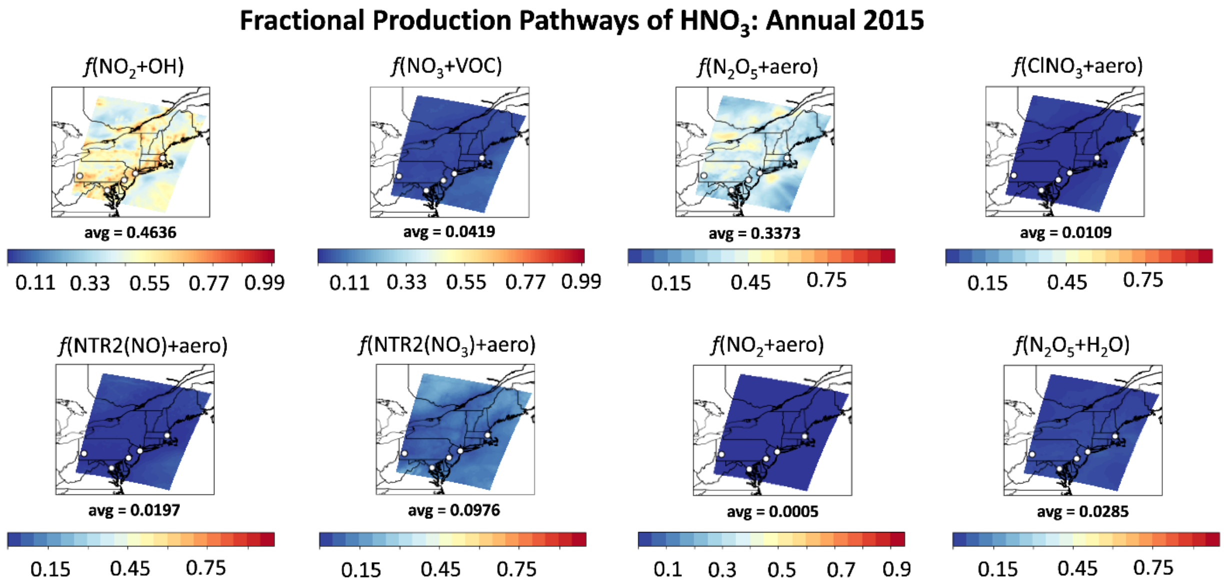

Fig. S4. The CMAQ model simulation of HNO_3 fractional production pathways for 2015 in the northeastern US domain for the bottom 1,000 m of the atmosphere. The open data points represent major urban areas, including (from north to south) Boston, MA, New York City, NY, Pittsburgh, PA, Philadelphia, PA, and Washington DC.

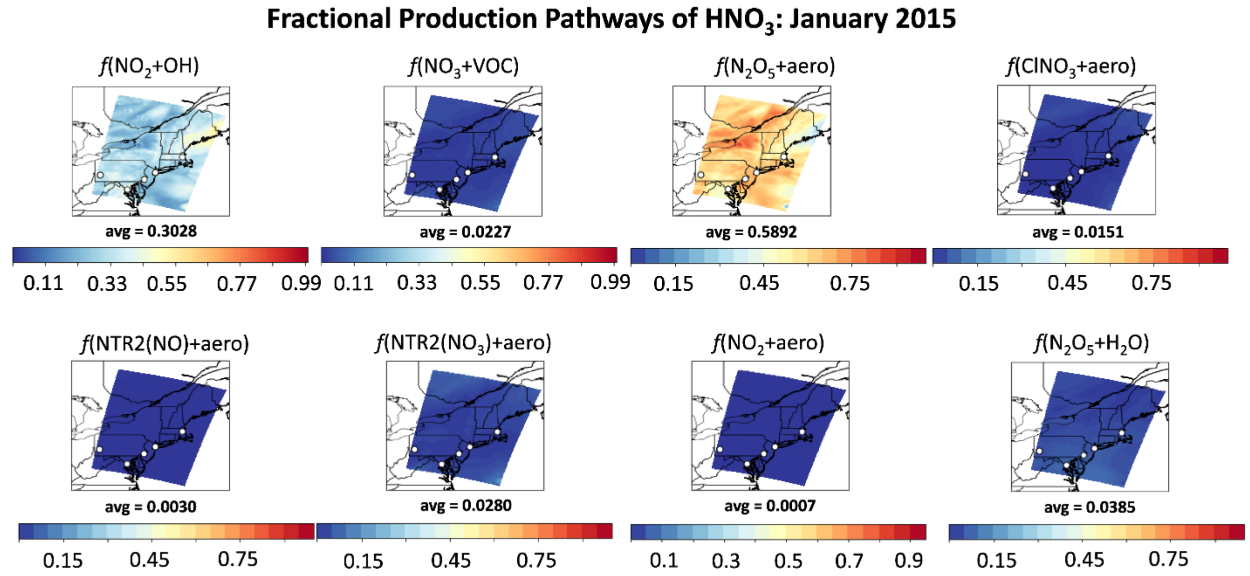

Fig. S5. The CMAQ model simulation of HNO_3 fractional production pathways for January 2015 in the northeastern US domain for the bottom 1,000 m of the atmosphere. The open data points represent major urban areas, including (from north to south) Boston, MA, New York City, NY, Pittsburgh, PA, Philadelphia, PA, and Washington DC.

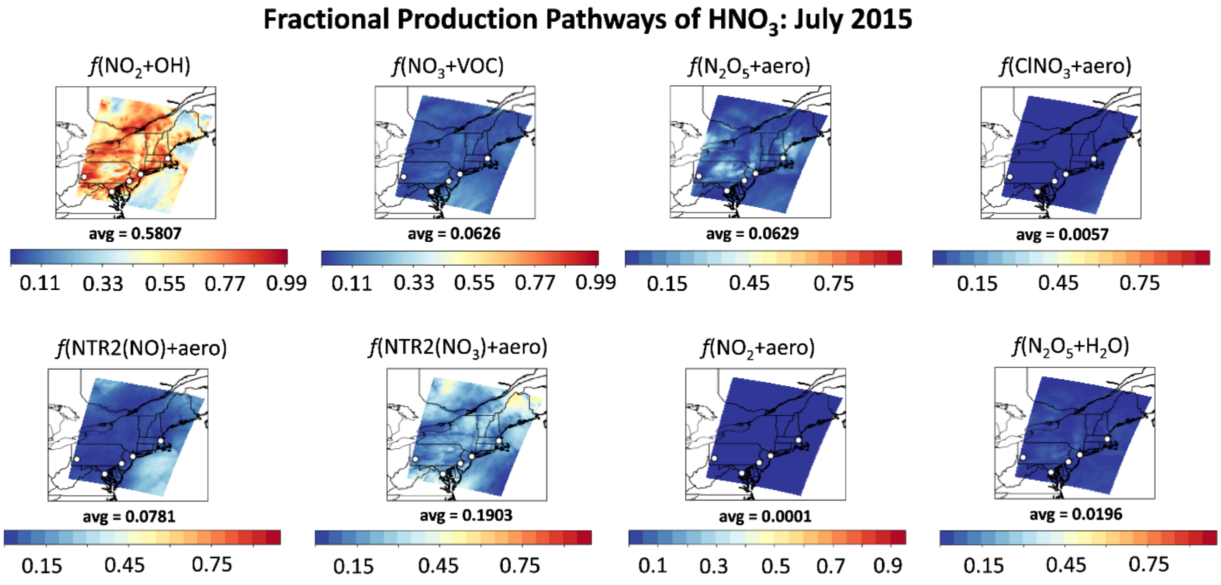

Fig. S6. The CMAQ model simulation of HNO_3 fractional production pathways for July 2015 in the northeastern US domain for the bottom 1,000 m of the atmosphere. The open data points represent major urban areas, including (from north to south) Boston, MA, New York City, NY, Pittsburgh, PA, Philadelphia, PA, and Washington DC.

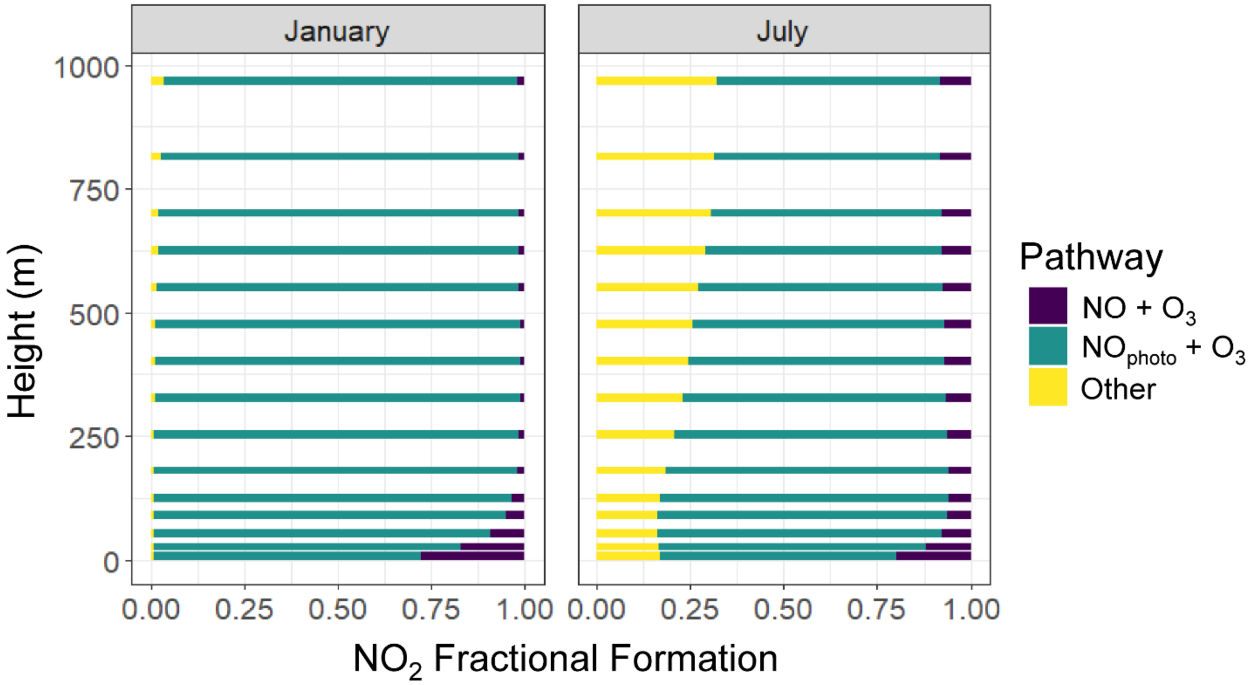

Fig. S7. The CMAQ model simulation of NO_2 fractional production pathways for January and July 2015 for Providence, RI, US (41.82 °N, 71.41 °W) as a function of atmospheric layer height. In total, the fifteen lowest vertical grill cells were considered, and the midpoint of each cell is indicated as the height.

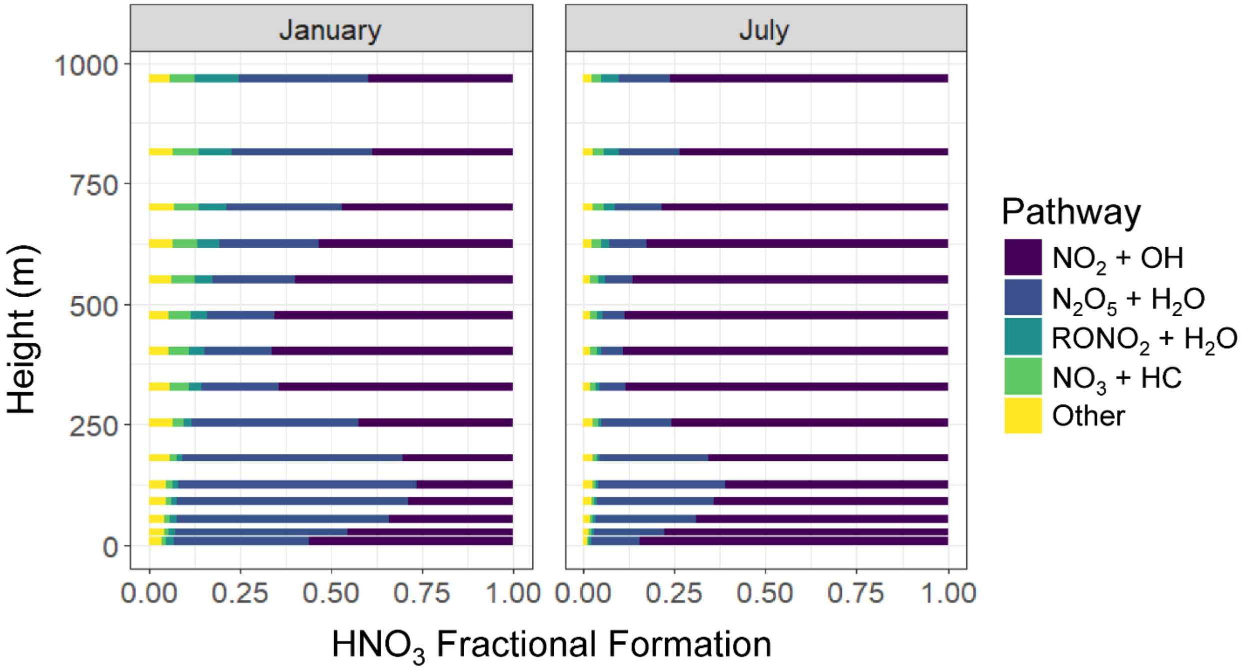

Fig. S8. The CMAQ model simulation of HNO_3 fractional production pathways for January and July 2015 for Providence, RI, US (41.82 °N, 71.41 °W) as a function of atmospheric layer height. In total, the fifteen lowest vertical grill cells were considered, and the midpoint of each cell is indicated as the height.

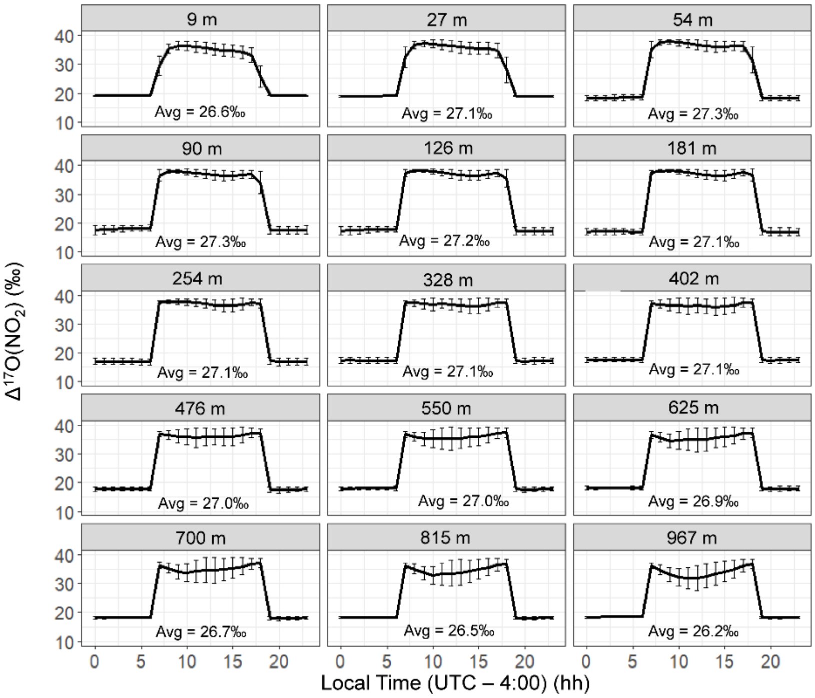

Fig. S9. Model simulations of the diel $\Delta^{17}O(NO_2)$ in Rumford, RI, using the CMAQ model for March 2015 averaged by hour. The $\Delta^{17}O(NO_2)$ calculations were conducted based on NO_2 production pathways for various atmospheric layer heights ~ 1,000 m, with the altitude mid-point of each vertical grid shown. The error bars correspond to the calculated standard deviations for each hour bin. The 24 h averaged (non-weighted) $\Delta^{17}O(NO_2)$ for each layer height is also indicated.

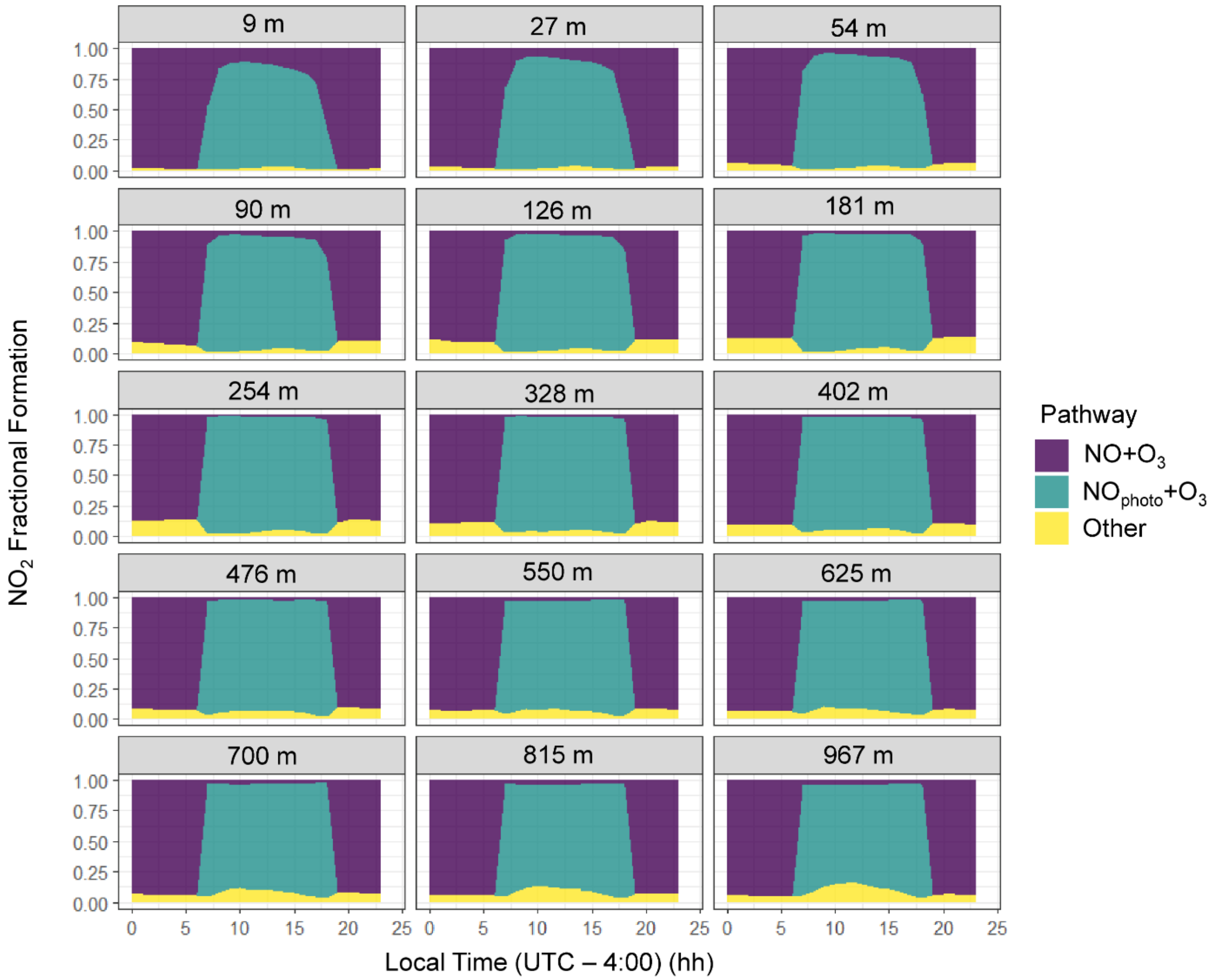

Fig. S10. Model simulations of the diel NO_2 fractional formation in Rumford, RI, using the CMAQ model for March 2015 averaged by hour. The model simulations were conducted for various atmospheric layer heights with the altitude mid-point of each vertical grid shown. The fractional formation pathways of NO_2 include NO + O_3, NO_{photo} + O_3 and all others (NO + RO_2, NO + HO_2, NO +ClO, NO_{photo}+RO_2, NO_{photo}+HO_2, NO_{photo}+ClO).

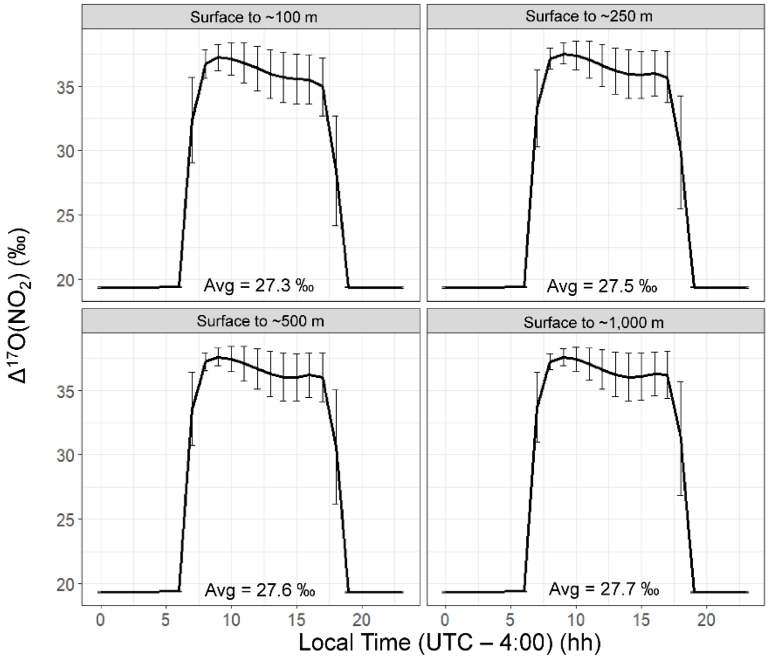

Fig. S11. Model simulations of the diel $\Delta^{17}O(NO_2)$ in Rumford, RI, using the CMAQ model for March 2015 averaged by hour. The $\Delta^{17}O(NO_2)$ was calculated based on the NO_2 production from the surface to various altitude bins, including up to ~100 m, ~250 m, ~500 m, and ~1,000 m. The error bars correspond to the calculated standard deviations for each hour bin. The 24 h averaged (non-weighted) $\Delta^{17}O(NO_2)$ for each altitude bin is also indicated.

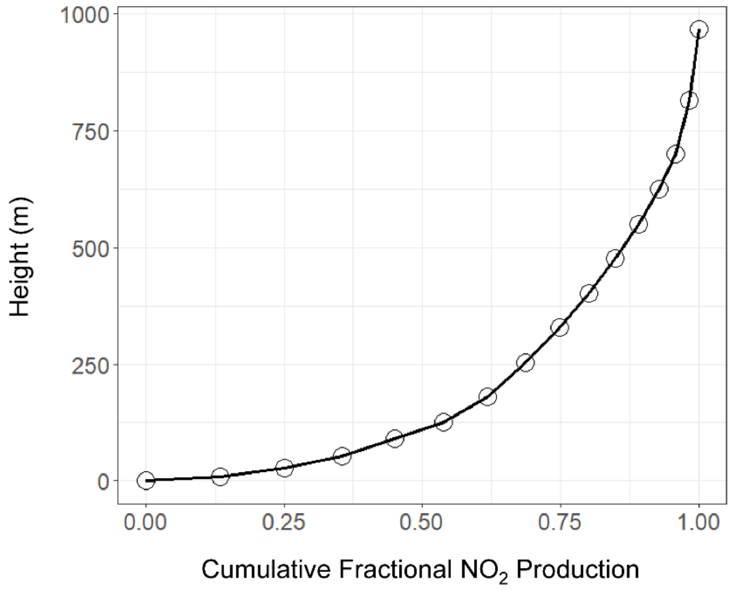

Fig. S12. Modeled cumulative fractional NO_2 production as a function of height up to ~1,000 m in Rumford, RI, using CMAQ for March 2015.

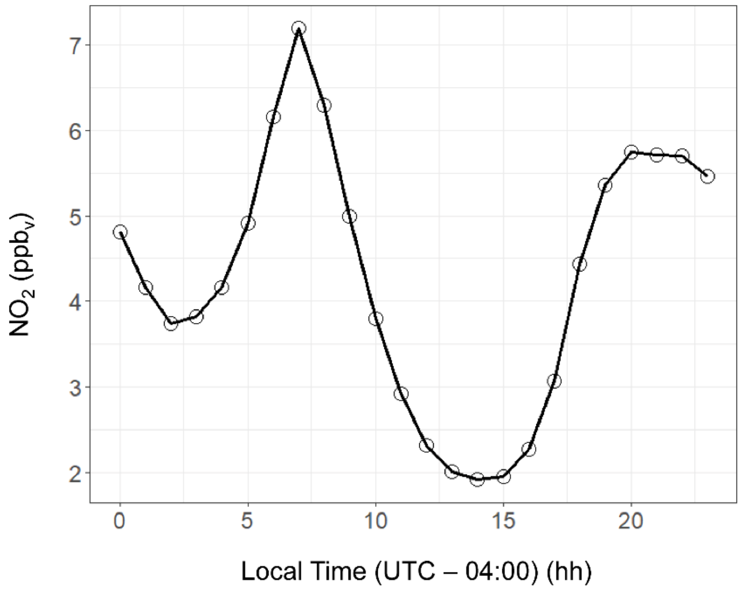

Fig. S13. Modeled average NO_2 mixing ratio from the surface to ~ 100 m in Rumford, RI, using CMAQ for March 2015 binned by hour.
